# Transposons and pathogenicity in *Xanthomonas*: acquisition of murein lytic transglycosylases by Tn*Xax1* enhances *Xanthomonas citri* subsp. *citri* 306 virulence and fitness

**DOI:** 10.7717/peerj.6111

**Published:** 2018-12-19

**Authors:** Amanda C.P. Oliveira, Rafael M. Ferreira, Maria Inês T. Ferro, Jesus A. Ferro, Mick Chandler, Alessandro M. Varani

**Affiliations:** 1 School of Agricultural and Veterinarian Sciences—Agricultural and Livestock Microbiology Graduation Program, Universidade Estadual Paulista, Jaboticabal, Sao Paulo, Brazil; 2 School of Agricultural and Veterinarian Sciences, Universidade Estadual Paulista, Jaboticabal, Sao Paulo, Brazil; 3 Department of Biochemistry, Georgetown University, WA, USA

**Keywords:** Lateral gene transfer, Comparative genomics, Biofilm, Xanthan gum, Tn*3*

## Abstract

*Xanthomonas citri* subsp. *citri* 306 (XccA) is the causal agent of type A citrus canker (CC), one of the most significant citriculture diseases. Murein lytic transglycosylases (LT), potentially involved in XccA pathogenicity, are enzymes responsible for peptidoglycan structure assembly, remodeling and degradation. They directly impact cell wall expansion during bacterial growth, septum division allowing cell separation, cell wall remodeling allowing flagellar assembly, bacterial conjugation, muropeptide recycling, and secretion system assembly, in particular the Type 3 Secretion System involved in bacterial virulence, which play a fundamental role in XccA pathogenicity. Information about the XccA LT arsenal is patchy: little is known about family diversity, their exact role or their connection to virulence in this bacterium. Among the LTs with possible involvement in virulence, two paralogue open reading frames (ORFs) (one on the chromosome and one in plasmid pXAC64) are passenger genes of the Tn*3* family transposon Tn*Xax1*, known to play a significant role in the evolution and emergence of pathogenicity in *Xanthomonadales* and to carry a variety of virulence determinants. This study addresses LT diversity in the XccA genome and examines the role of plasmid and chromosomal Tn*Xax1* LT passenger genes using site-directed deletion mutagenesis and functional characterization. We identified 13 XccA LTs: 12 belong to families 1A, 1B, 1C, 1D (two copies), 1F, 1G, 3A, 3B (two copies), 5A, 6A and one which is non-categorized. The non-categorized LT is exclusive to the *Xanthomonas* genus and related to the 3B family but contains an additional domain linked to carbohydrate metabolism. The categorized LTs are probably involved in cell wall remodeling to allow insertion of type 3, 4 and 6 secretion systems, flagellum assembly, division and recycling of cell wall and degradation and control of peptidoglycan production. The Tn*Xax1* passenger LT genes (3B family) are not essential to XccA or for CC development but are implicated in peptidoglycan metabolism, directly impacting bacterial fitness and CC symptom enhancement in susceptible hosts (e.g., *Citrus sinensis*). This underlines the role of Tn*Xax1* as a virulence and pathogenicity-propagating agent in XccA and suggests that LT acquisition by horizontal gene transfer mediated by Tn*Xax1* may improve bacterial fitness, conferring adaptive advantages to the plant-pathogen interaction process.

## Introduction

The *Xanthomonadaceae* includes some of the major Gram-negative phytopathogens worldwide (Mansfield et al., 2012). Among them, *Xanthomonas citri* subsp. *citri* strain 306 (XccA) stands out as the causal agent of type A citrus canker (CC), one of the most significant citriculture diseases (Da Silva et al., 2002; Graham et al., 2004). This leaf-spotting and fruit rind-blemishing disease induces hyperplasic and hypertrophic lesions surrounded by water-soaked margins and a yellow halo on leaves, stems and fruits (Jalan et al., 2013). These lesions may cause fruits and leaves to drop prematurely. Disease development relies on several virulence factors and host range determinants encoding an intricate number of plant-pathogen interaction mechanisms.

XccA-host interaction depends on expression of a number of bacterial and plant host genes (Ference et al., 2018). Extracellular polysaccharides, lipopolysaccharides, cell wall degradation enzymes, effectors and their secretion systems, two-component regulation systems (TCS) and cell-to-cell signaling or quorum-sensing (QS) are the main XccA-encoded virulence and pathogenicity factors (Jalan et al., 2014). In addition, a number of other virulence factors, such as cell motility, detection of cell-wall-acting antibiotics, and other bacterial physiological processes also rely on bacterial mechanisms dependent on cell wall synthesis, remodeling and degradation. The totality of these processes determines XccA infection dynamics.

Peptidoglycan cleavage and subsequent remodeling is accomplished by murein lytic transglycosylases (LTs) (Holtje, 1998). Therefore, the LTs are presumably involved with many of the XccA virulence and pathogenicity factors, but also seem to play several other roles in cell function: for instance, cell wall expansion during bacterial growth; septum division allowing cell separation; cell wall remodeling allowing flagellar assembly; bacterial conjugation; muropeptide recycling and cell wall lysis allowing sporulation and germination of Gram-positive bacteria (Dik et al., 2017). LTs are classified into six families (for a full review see Dik et al. (2017)) according to predicted function and domain distribution, although the function and activity of a number of LTs remains to be determined. A true appreciation of LT diversity and distribution has been hampered by a lack of accurate annotation in publicly available genome sequences.

Information on the XccA LT arsenal, in particular, is limited. Previous studies had investigated the plasmid pXAC64 LT copy (*XAC_RS22275*; *mltB2*.1) in XccA using functional assays. The results suggested a role in XccA interaction with the citrus host (Laia et al., 2009). *XAC_RS22275* has a paralogue copy, *XAC_RS16355* (*mltB2*.2), situated on the XccA chromosome, with 99% identity to the plasmid copy. Both copies are passenger genes of derivatives of a Tn*3* family transposon, Tn*Xax1*. The Tn*3* family is a widespread group of transposable elements containing one or more passenger genes in addition to their transposition genes (see Nicolas et al. (2015) for review). Tn*3* family transposons are also capable of mediating gene reassortment and genomic plasticity in their host genomes (Nicolas et al., 2015). Tn*Xax1* includes transposase (*tnpA*), resolvase (*tnpS*) and recombinase (*tnpT*) genes involved in the transposition process in addition to the *mltB2* and other virulence determinants. The Tn*Xax1* chromosome copy, Tn*Xax1.2*, contains additional passenger genes coding for Type 3 Secretion System (T3SS) effector proteins XopE3 and XopAI, while the Tn*Xax1.1* plasmid copy contains the XopE2 effector (Ferreira et al., 2015).

Previous studies demonstrated that the Tn*Xax1-*like element is conserved in *Xanthomonadales* and plays a significant role in evolution and emergence of pathogenicity in this order (Ferreira et al., 2015; Gochez et al., 2018).

Although LTs are involved in cell-wall synthesis, remodeling and degradation, their exact role in pathogenicity in XccA is not entirely clear. In silico analysis coupled with functional assays of those proteins might shed some light about their role in XccA-mediated CC and virulence.

In the results reported here we identified, annotated, and performed comparative analysis of the murein LTs carried by XccA and also experimentally explored the function of the LTs carried by the Tn*Xax1* transposon. To investigate association between horizontal gene transfer and the plant-pathogen interaction virulence process, we generated site-directed deletion mutants of Tn*Xax1.1* (*XAC_RS22275*) and Tn*Xax1*.2 (*XAC_RS16355*) LT passenger genes in various combinations and examined the effects on a number of properties associated with virulence and pathogenicity.

## Materials and Methods

### Identification and in silico analysis of LTs in *Xanthomonas citri*

The search for LT genes was performed on the GenBank database at NCBI using the BLAST tool (Altschul et al., 1997) and catalogued using ClustalX (Larkin et al., 2007) and *InterProScan* (Finn et al., 2017) tools using the classification proposed by Dik et al. (2017).

### Bacterial strains and growth media

Strains used in this study are listed in Table S1. The *X. citri* subsp. citri wild-type strain 306 (XccA) and mutant strains were cultivated in nutrient broth (NB: 0.5% peptone, 0.3% beef extract), nutrient agar (NA: 0.5% peptone, 0.3% beef extract, 1.5% agar), XVM2 (20 mM NaCl, 10 mM (NH4)2SO_4_, five mM MgSO_4_, one mM CaCl_2_, 0.16 mM KH_2_PO_4_, 0.32 mM K_2_HPO_4_, 0.01 mM FeSO_4_, 10 mM fructose, 10 mM sucrose, 0.03% casaminoacids, pH 6.7), gum media (2.5% glucose, 3% yeast extract, 2% K_2_HPO_4_, 0.1 g/L MgSO_4_.7H_2_O), tryptone swimming broth (1% tryptone, 0.5% NaCl, 0.3% agar), tryptone swarming broth (1% tryptone, 0.5% NaCl, 0.7% agar) and SB (0.5% sucrose, 0.5% yeast extract, 0.5% peptone and 0.1% glutamic acid, pH 7.0) at 29 °C. *Escherichia coli* strains were cultivated in Luria-Bertani medium (LB: 0.1% tryptone, 0.05% yeast extract, 0.10% NaCl, 1.5% agar; pH 7) at 37 °C. Antibiotics for selection of recombinant plasmids were used as needed at the following concentrations: kanamycin (Kn), 50 μg/mL; carbenicillin (Carb), 50 μg/mL; spectinocycin (Spc), 50 μg/mL.

### Generation of *XAC_RS16355* (*mltB2*.2) and *XAC_RS22275* (*mltB2*.1) ORF deletion mutants

Site-directed mutagenesis through overlap extension PCR and homologous recombination (Lee et al., 2004) was used to generate three different mutants. PCR and ligations were performed as shown in Fig. S1, using XccA bacterial genomic DNA as template and primers described in Table 1, in order to obtain deletion mutants *ΔmltB2-pXAC64* (*ΔmltB2*.1), *ΔmltB2*—chromosome copy (*ΔmltB2*.2), and the double mutant, *ΔmltB2*-pXAC64—*ΔmltB2* (*ΔmltB2*.1–*ΔmltB2*.2). Primers B and C, R1 and F2 were designed with self-complementary tails (Table 1). PCR was first performed to amplify A/B and C/D, F1/R1 and F2/R2, followed by a ligation PCR using primers B/C and F1/R2, together with first PCR products as templates (A/D and F1/R2). This resulted in deletion of the entire selected genes (Fig. S1). Final PCR products A/D and F1/R2 were double digested with NheI/HindIII and BamHI/ApaI (New England BioLabs Inc®, Ipswich, MA, USA), respectively, according to the manufacturer’s instructions (Fig. S1) and the DNA fragments with the *mltB2* deletion were cloned into suitable similarly digested vectors. The DNA sequence of each mutant clone was verified.

**Table 1:** Primers used in this work.

ORF	Oligonucleotide name	Sequence (5′–3′)	Restriction site	Product (bp)	Purpose
*XAC_RS16355* (*mltB2*.2)	A (F)**	CATA**GCTAGC**GGTTCGGTGACTACACCTTG	NheI**	237	Site directed mutagenesis
	B (R)*	**CATCCCCGAGAGATACCC**GAGGTGGTG	–		
	C (F)*	**GGGTATCTCTCGGGGATG**TACGTCGGA	–	624	Site directed mutagenesis
	D (R)**	GACGCTTTCTTCTGGCTG**AAGCTT**CTGT	HindIII**		
	F1 (F)**	GTGT**GGATCC**GGGCATTCTGACCGCCAT	BamHI**		
*XAC_RS22275* (*mltB2*.1)	R1 (R)*	**GCTCGGTGATTTTCCGTCT**GCAGGATCT	–	181	Site directed mutagenesis
	F2 (F)*	**GACGGAAAATCACCGAGCT**GCAGCAGAG	–	198	Site directed mutagenesis
	R2 (R)**	AAGGTGAGGGATATCCCCGATCAGG**GGGCCC**GC	ApaI**		
*XAC_RS16355* (*mltB2*.2)	XAC_RS16355 (R)	AAGGGGAGGCGCGTCCTCTTGGCG	–	600	Sequencing
*XAC_RS22275* (*mltB2*.1)	XAC_RS22275 (R)	TGGGGCCGATATCGATAAAAAAAGGAATCC	–	600	Sequencing

**Notes:**

B/C and R1/F2 oligonucleotides blue sequences are complementary to sequences in red of its pairing primer.

* F, Forward; R, Reverse. Oligonucleotides synthesized by SIGMA-ALDRICH BRASIL.

** Underlined regions represent added restriction sites.

pOK1 and pNPTS138 suicide vectors (Kaniga, Delor & Cornelis, 1991) were used to introduce the deletions into *mltB2*.1 and *mltB2*.2 genes, respectively, through homologous recombination. Ligation between vectors and fragments carrying the deletion mutants was performed with T4 DNA Ligase (New England BioLabs Inc®) using the manufacturer’s specifications. Once ligated, closed vectors were transformed into chemically competent DH10B *E. coli* (Sambrook, Fritschi & Maniatis, 1989) and transformant colonies were selected using suitable antibiotics (kanamycin for pNPTS138 and spectinomycin for pOK1). DNA from selected colonies was sequenced to confirm successful deletion.

A confirmed pNPTS138-*ΔmltB2*.2 clone was used to generate the chromosomal Δ*mltB2*.2 mutant and pOk1-Δ*mltB2*.1 clone to generate the Δ*mltB2*.1 pXAC64 mutant by electroporation of electrocompetent XccA cells (Do Amaral et al., 2005). Following transformation with antibiotic selection, the transformed cells were selected by susceptibility to sucrose provided by the suicide vector in NA medium. After loss of the suicide vector, the confirmed mutants are no longer resistant to antibiotics (kanamycin for pNPTS138 and spectinomycin for pOK1) nor susceptible to sucrose in NA medium. The double mutant, *ΔmltB2*.1–*ΔmltB2*.2, was obtained using the confirmed pNPTS138-*ΔmltB2*.1 clone to transform electro-competent Δ*mltB2*.2 cells, following the previous selection step. Mutant confirmation was performed with PCR followed by DNA sequencing on 3730xI DNA analyzer (Thermo Fisher Scientific, Waltham, MA, USA) using primers with high specificity to each gene (*XAC_RS16355*-R and *XAC_RS22275*-R) (Table 1).

### *In planta* pathogenicity tests

Two methods were used for assessing pathogenicity. In the first, the strains were inoculated onto the leaf surface by the spray method (Granato et al., 2016; Yan & Wang, 2011). All strains were cultured for 48 h on NA plates at 29 °C, followed by inoculation on NB until a final O.D. 600 nm of around 0.5–0.8 ABS. Cells were collected by centrifugation and resuspended in PBS buffer to an O.D. 600 nm of 0.3, equivalent to 10^8^ CFU (Colony Forming Unit)/mL. Four different “Pera Rio” orange trees (*Citrus sinensis* L. Osbeck) were sprayed with each bacterial suspension until all leaves were completely coated, then covered with a clear plastic bag for 24 h. All leaves presenting CC symptoms were photographed 20 days after inoculation (DAI). Only young orange tree leaves (around 30 days old) were used for all experiments. Inoculated plants were kept in a high efficiency particulate arrestance filtered plant laboratory with controlled environmental conditions (28–30 °C, 55% humidity, 12 h light cycle).

In the second method, a bacterial suspension was injected directly into young leaves (Laia et al., 2009). Mutant and wild-type (WT) XccA strains were cultivated in 10 mL NB for 16 h at 29 °C and then centrifuged at 3,000 × *g* for 12 min at room temperature. The supernatant was discarded and the pellets resuspended in autoclaved tap water to an O.D. 600 nm of 0.3 ABS, equivalent to 10^8^ CFU/mL. These inocula were infiltrated at two points on the underside of three young leaves (technical replicates) in three different “Pera Rio” orange (*C. sinensis* L. Osbeck) plants (biological replicates) using needleless hypodermic syringes. WT XccA was infiltrated on each leaf on the left-hand side of the central vein, while mutants were infiltrated on the right-hand side, so that symptom progression could be compared side by side. One leaf in each plant was infiltrated with sterile distilled water as negative control.

### *Ex planta* growth curve assay

Mutant strains and WT XccA were cultivated on NA for 24 h at 29 °C and individually transferred to 20 mL NB for 16 h at 29 °C (final O.D. 600 nm around 0.5–0.8 ABS). Cells were collected by centrifugation and resuspended in fresh NB to an O.D. 600 nm of 0.1 in a final volume of 1.5 mL. The inoculum was incubated in a Synergy H1 microplate reader (BioTek®, Winooski, VT, USA) under constant agitation at 29 °C and automated O.D. readings taken every 30 min. Growth curves were generated using *Graphpad Prism* 6 software, based on three technical and three biological replicates (Lacerda et al., 2017).

### Viable cell count after plating on SB and NB media

All strains were cultured for 48 h on NA plates at 29 °C, followed by inoculation into NB or SB medium to a final O.D. 600 nm of around 0.5–0.8 ABS. Cells were diluted to an O.D. 600 nm of 0.3, equivalent to 10^8^ CFU/mL and 200 μL of each suspension was inoculated into 50 mL sterile falcon tubes containing 10 mL of each media. Cultures were kept at 29 °C under agitation (180 rpm) for 24 h, at which point O.D. readings were taken to ensure that all cultures were at the same O.D. Samples were diluted and 50 μL were plated on NA or SB agar. Plates were incubated for 72 h. All sample counts were based on three biological replicates.

### *In planta* growth curve assay

*In planta* growth curve assays were performed on mutants and WT XccA according to Laia et al. (2009) with modifications. Strains were cultivated in NA for 48/72 h at 29 °C. Cells from the surface of the plates were inoculated into fresh NB and grown to a final O.D. 600 nm of around 0.5−0.8 ABS. Cells were collected by centrifugation and ressuspended in falcon tubes containing 50 mL of autoclaved Milli-Q water to an O.D. 600 nm of 0.3, equivalent to 10^8^ CFU/mL. This inoculum was diluted 100-fold (10^6^ CFU/mL) and infiltrated on the underside of three young leaves (technical replicates) in three different plants (biological replicates) of “Pera Rio” orange (*C. sinensis* L. Osbeck) using hypodermic syringes. The number of cells per leaf area was determined at intervals by counting isolated colonies on NA plates using a microculture technique (Laia et al., 2009) (three dilutions and in triplicate).

### Biofilm formation study

Assays were performed in rich NB medium (Yan, Hu & Wang, 2012) and defined virulence inducing media XVM2 supplemented with 1% w/v glucose (Rigano et al., 2007). Once strains were cultivated on NA, as already described, log phase cells were transferred to rich NB/defined XVM2 media and ressuspended at 10^8^ CFU/mL. One mL of the bacterial suspension was then transferred to borosilicate glass tubes and incubated without agitation for 96 h at 29 °C. Biofilm formation was visualized with 0.1% crystal violet staining followed by washing with ultrapure sterile water. The remaining stained cells were dissolved in one mL 95% ethanol and quantified at O.D. 595 nm in a Bio-Rad iMark spectrophotometer. Averages of four technical and biological replicates were used to compare biofilm production in each strain.

### Cell aggregation assays

Mutant and WT XccA strains were cultivated in NB for 16 h at 29 °C. Cell cultures were adjusted to 0.3 at O.D. 600 nm to a final volume of 10 mL per culture, and then two mL (pre-inoculum) were transferred to sterile 50 mL Erlenmeyer flasks containing 25 mL of NB. Once O.D. 600 nm reached 2.0 ABS, 10 mL of the cultures were transferred to borosilicate tubes. All cultures were vigorously agitated for 15 s and a 100 μL aliquot (0 h) was taken from each tube one cm below the top of each culture surface and added to 900 μL of fresh media (10× dilution). O.D. 600 nm was measured and multiplied by 10-fold to determine the cell density in the original culture. All tubes remained static during the entire experiment and samples were collected at hourly intervals as already described. Averages of two technical and four biological replicates were used to compare cell aggregation in each strain.

### Assay for xanthan gum production

Mutant and WT XccA strains were cultivated on NA for 48 h at 29 °C and individually transferred to 10 mL NB for 16 h at 29 °C, 180 rpm agitation. The O.D. 600 nm was adjusted to 0.3 (10^8^ CFU/mL) in a final volume of 2.5 mL. The inoculum was transferred to 250 mL Erlenmeyer flasks containing 100 mL gum media (25 g/L glucose, three g/L yeast extract, two g/L K_2_HPO_4_, 0.1 g/L MgSO_4_.7H_2_O) and incubated at 29 °C, 180 rpm for 96 h (Shu & Yang, 1990).

The cells from 96 h cultures were centrifuged at 9,666×*g* for 40 min at 4 °C. The bacterial pellets were resuspended in one mL autoclaved milli-Q water, transferred to a pre-weighed beaker and weighed again after 24 h at 70 °C. The supernatants were transferred to 500 mL beakers. The gum was recovered from the supernatants by adding four g of KCl to each beaker, followed by stirring at room temperature for 15 min. Two volumes of cold ethanol were added and the xanthan gum from each beaker was removed to pre-weighed plastic containers with the aid of a glass rod and a plastic sieve. After 72 h at 37 °C the plastic containers were weighed again and the gum amount was calculated. All extractions were performed with three biological replicates.

### Bacterial motility assay

Swimming motility was assayed (Raetz & Whitfield, 2002) using plates of tryptone swimming broth with 0.3% added agar, while the swarming motility assay was performed on plates of tryptone broth with 0.7% added agar. Plates were incubated at 29 °C for 96 h with no agitation. Cell motility disks formed by the colonies on the surface of the plates were photographed by a digital immobilized camera and measured using ImageJ software. Assays were performed using five technical and four biological replicates.

### Statistical analysis

All results obtained were analyzed with Sigma plot software, using Tukey’s test to compare means at 5% or 1% probability. Cell aggregation assay results were compared with ANOVA one-way tests.

### Plasmid pXAC64 copy number determination

The plasmid copy number was estimated from data available from the NCBI Sequence Read Archive (SRA) (accession numbers SRX195367 and SRX195344) from the BioProject PRJNA177640. XccA raw reads were downloaded and mapped to the XccA genome (NC_003919, NC_003922 and NC_003921) with bowtie2 with—*very-sensitive* and—*end-to-end* parameters (Langmead & Salzberg, 2012). The average coverage depth of the XccA chromosome and pXAC64 plasmid was estimated with SAMtools *depth* (Li et al., 2009). The pXAC64 copy number was determined by dividing the coverage depth of the chromosome by that of the plasmids.

## Results

### LT identification, diversity and categorization in the XccA genome

We identified 13 LTs in the XccA genome. A total of 12 belong to subfamilies 1A, 1B, 1C, 1D (two copies), 1F, 1G, 3A, 3B (two copies), 5A, 6A and one non-categorized LT gene (Table 2). According to alignments, protein domain analyses, literature searches and annotation, the categorized LTs are probably involved in cell wall remodeling to allow assembly of type 3, 4 and 6 secretion systems into the cell wall, flagellum assembly, division and recycling of cell wall material, and degradation and control of peptidoglycan production (Table 2).

**Table 2:** LTs and biosynthetic peptidoglycan transglycosylase found in *Xanthomonas citri subsp. citri*.

Locus tag	Gene name	Coordinates	Length (aa)	LT classification	Proposed cellular role in XccA*
XAC_RS18005	*slt*	4,223,769..4,225,742	657	1A	Cell-wall recycling/peptidoglycan monomer production
XAC_RS12500	*mltC*	2,862,053..2,863,036	327	1B	Peptidoglycan degradation
XAC_RS11470	*mltE*	2,639,080..2,639,691	203	1C	Insertion of the type VI secretion system
XAC_RS13860	*mltD2*	3,190,523..3,192,106	527	1D	Unknown (related to plant-bacteria recognition in bacterial pathogenesis)
XAC_RS05550	*mltD1*	1,241,249..1,242,427	392		
XAC_RS13315	*sltF*	3,082,452..3,083,294	280	1F	Flagellum assembly
XAC_RS02185	*hpaH/etgA*	487,771..488,334	187	1G	Insertion of the type III and IV secretion system
XAC_RS03435	*mltB*	785,110..786,249	379	3A	Cell-wall recycling and resistance
XAC_RS16355	*mltB2.2*	3,798,986..3,800,263	425	3B	Related to XccA virulence and fitness
XAC_RS22275	*mltB2.1*	3,862..5,088 (pXAC64)			
XAC_RS05780	*mltG*	1,281,557..1,282,630	357	5A	Regulates peptidoglycan strand length
XAC_RS03440	*rlpA*	786,246..787,673	475	6A	Cell division and/or morphogenesis
XAC_RS21660	n/a	5,077,186..5,079,348	828	Unclassified/related to 3B	Carbohydrate metabolic process/peptidoglycan binding function
XAC_RS15470	*mtgA*	3,564,980..3,565,720	246	Biosynthetic peptidoglycan transglycosylase	Peptidoglycan biosynthesis

**Notes:**

*citri* 306 genome and pXAC64 plasmid classification and proposed gene names and role in XccA (for a detailed alignment and domain analysis please see Fig. S2).

* Considering that all classified LTs has a general function related to the rearrangement of the peptidoglycan layer.

The non-categorized LT (*XAC_RS21660*) is exclusive to the *Xanthomonas* genus. It is related to the 3B subfamily, but contains an additional glycoside hydrolase-type carbohydrate-binding domain (IPR014718) linked to carbohydrate metabolism (Fig. S2). It is almost twice the size in amino acid residues compared to other 3B subfamily members and has low global sequence identity. However, it shares the peptidoglycan binding-like (IPR002477) and transglycosylase SLT 2 (IPR031304) domains, suggesting a potential role related to that of the other 3B family genes, *mltB2*.1 and *mltB2*.2, found in XccA.

In addition, we also identified a peptidoglycan transglycosylase (*mtgA*) which catalyzes peptidoglycan biosynthesis, and is widely distributed in gamma-proteobacteria, leading us to believe that *mtgA* is essential for XccA.

The two XccA 3B subfamily *mltB2* genes (Dik et al., 2017) are both 425 amino acid residues long with a signal peptide of 27 aa. They are 99% identical in sequence, differing by four synonymous nucleotide substitutions and only one non-synonymous substitution (Ser from *mltB2*.1 to Ala from *mltB2*.2 at position 51). Each is carried by a Tn*3*-family transposon and therefore capable of horizontal transmission and we previously suggested that they are involved in XccA virulence (Ferreira et al., 2015, 2016) although their specific cellular role still remains unknown.

To investigate the effect of the Tn*Xax1 mltB2*.1 and *mltB2*.2 passenger LT genes on XccA virulence and pathogenicity, they were individually deleted (Materials and Methods) to generate the single mutants, *mltB2*.1-pXAC64/*ΔmltB2*.1 (*ΔmltB2*.1) and *mltB2*.2/Δ*mltB2*.2 (*ΔmltB2*.2), respectively. We also created the double mutant *ΔmltB2*.1–*ΔmltB2*.2 (Fig. S3).

### Tn*Xax1* associated LTs affect CC progression

**Figure 1:**
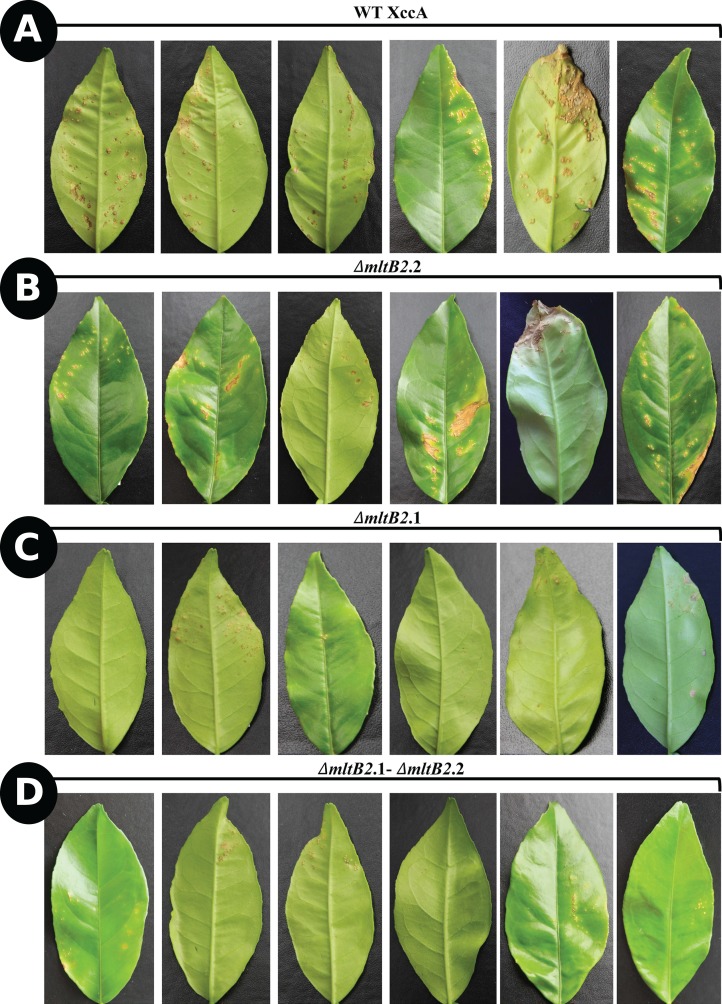
*In planta* pathogenicity test by spray method. CC disease progression among *ΔmltB2*.2 (B), *ΔmltB2*.1 (C) and *ΔmltB2*.1–*ΔmltB2*.2 (D) mutants compared to WT XccA (A), 20 days after inoculation. Each bacterial suspension was sprayed at 10^8^ CFU/mL onto a whole “Pera Rio” orange tree. Six leaves of each treatment with the highest disease severity were photographed.

To determine the effect of *ΔmltB2*.1, *ΔmltB2*.2 and *ΔmltB2*.1–*ΔmltB2*.2 mutations on XccA pathogenicity, inoculation by the spray method was used (Materials and Methods) on young leaves of the susceptible orange variety “Pera Rio” (*C. sinensis* L. Osbeck). After 20 days of growth, the WT XccA and *ΔmltB2*.2 mutant strains showed the highest numbers and size of canker lesions, while the *ΔmltB2*.1 and *ΔmltB2*.1–*ΔmltB2*.2 strains generated much smaller and fewer lesions (Fig. 1).

**Figure 2:**
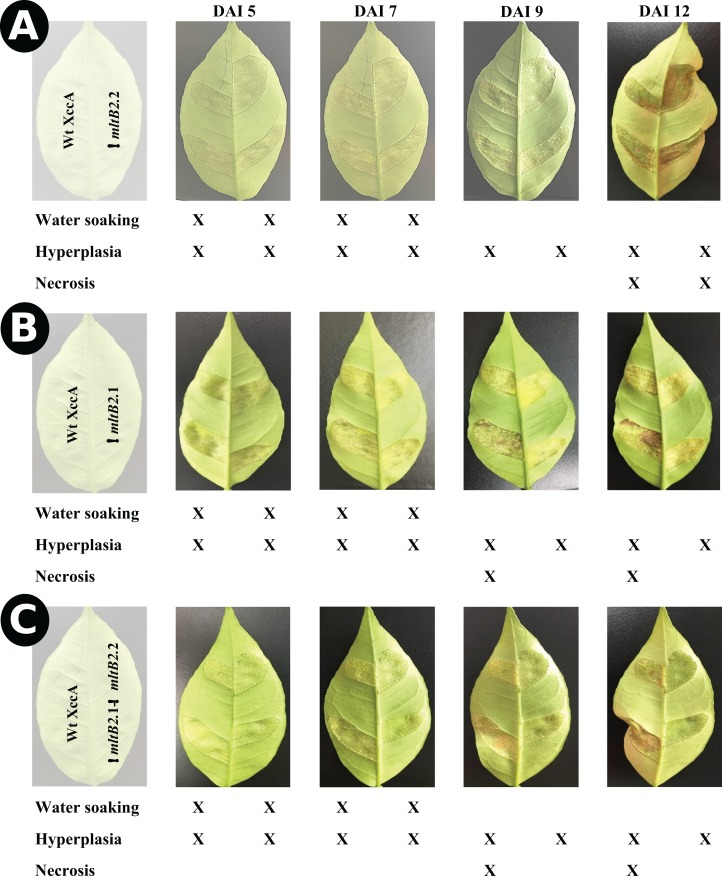
*In planta* pathogenicity test by injection directly into young leaves. CC disease progression among *ΔmltB2*.2 (A), *ΔmltB2*.1 (B) and *ΔmltB2*.1–*ΔmltB2*.2 (C) mutants over the course of 12 days. Inocula were infiltrated at 10^8^ CFU/mL in two points on the underside of three young leaves (technical replicates) in three different plants (Biological replicates) of “Pera Rio” orange (*Citrus sinensis* L. Osbeck) using hypodermic syringes. On each leaf WT XccA was infiltrated on the left-hand side of the central vein, while mutants were infiltrated on the right-hand side. Table indicates symptom progression variation among mutants over the course of days. Degrees of disease severity are represented by water soaking, hyperplasia and necrosis of tissue, culminating with the spread of the pathogen to the environment. *ΔmltB2*.1 and *ΔmltB2*.1–*ΔmltB2*.2 mutants didn’t show any signs of necrosis at day 12, while necrosis was clearly present on *ΔmltB2*.2 and XccA.

To confirm this, the more aggressive “inoculation” procedure was used (Materials and Methods) with similar results. Photographs of typical leaves as a function of DAI are shown in Fig. 2.

The results indicate that the effects of the plasmid and chromosomal *mltB2* mutations are quantitatively different. The *ΔmltB2*.2 chromosomal mutant shows only a small or no effect compared to the WT XccA, whereas the mutant strains carrying the plasmid *ΔmltB2*.1 mutation (*ΔmltB2*.1 and the double mutant *ΔmltB2*.1–*ΔmltB2*.2) generate fewer and smaller canker lesions.

The effects of the *mlt* mutations on canker formation might reflect a direct role in pathogenicity, an indirect effect such as enhancement of cell growth and/ or survival *in planta* or a combination of these effects. To address these possibilities, we performed a variety of analyses on different aspects of bacterial cell growth.

### mltB2.1 and *mltB2*.2 mutations do not affect growth *ex planta*

**Figure 3:**
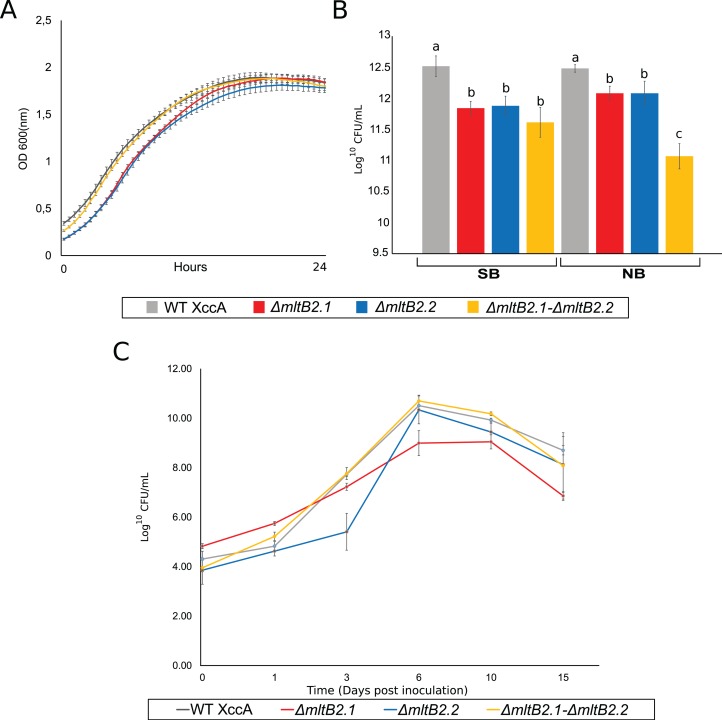
*Ex planta* bacterial growth, *in planta* bacterial growth and plating on NB and SB. (A) *Ex planta* bacterial growth curve performed on rich medium NB for 48 h. (B) Viable cell count on NB and SB medium depicting bacterial survival after plating. (C) *In planta* bacterial growth curve. Error bars indicate the standard error of three independent biological and technical replicates.

The most direct explanation for the effect of *mltB2* is that the genes enhance bacterial host cell growth *ex planta*. The results of simple growth curves of the WT and the three mutant configurations carried out in NB medium and followed by OD600 measurements is shown in Fig. 3A. All cultures exhibit very similar growth reaching stationary phase after about 15 h.

The effects of the mutant *mltB2* alleles are therefore unlikely to be directly linked to *ex planta* cell growth per se, since the growth curves of the WT XccA and the mutants appeared nearly identical. This also shows that *mltB2* is not essential for XccA growth. However, since MltB2 functions may be involved in cell wall metabolism, it is possible that, while cell mass (as measured by O.D. 600) is not affected during growth in liquid culture, the genes may provide protection to the host cells under other conditions.

### *mltB2*.1 and *mltB2*.2 are not essential for *in planta* bacterial survival, and may provide protection to plating while also intervening with surface cell attachment

To address the possibility that *mltB2* plays a more subtle role and could provide protection when the bacteria are located on solid surfaces, such as agar plates, cultures grown for 24 h at 29 °C in NB or SB medium were plated on the corresponding agar plates. Viable cell count (log^10^ CFU/mL) are shown in Fig. 3B. Surprisingly, although the XccA WT and mutant cultures grew to the same OD in a given medium, the mutants exhibited a reduced number of CFU/mL compared to the WT strain in both NB and SB media, implying either a sensitivity to plating with the MltB2 defect and/or a difference in cell size, or other physiological processes affected by MltB2.

In spite of this, the single and double mutants are all able to propagate *in planta* (Fig. 3C). To further examine the behavior of the mutant strains we investigated a number of their growth and virulence-related properties.

### *mltB2*.1 and *mltB2*.2 have only a moderate impact on biofilm formation and xanthan gum production but none on cell aggregation and motility

**Figure 4:**
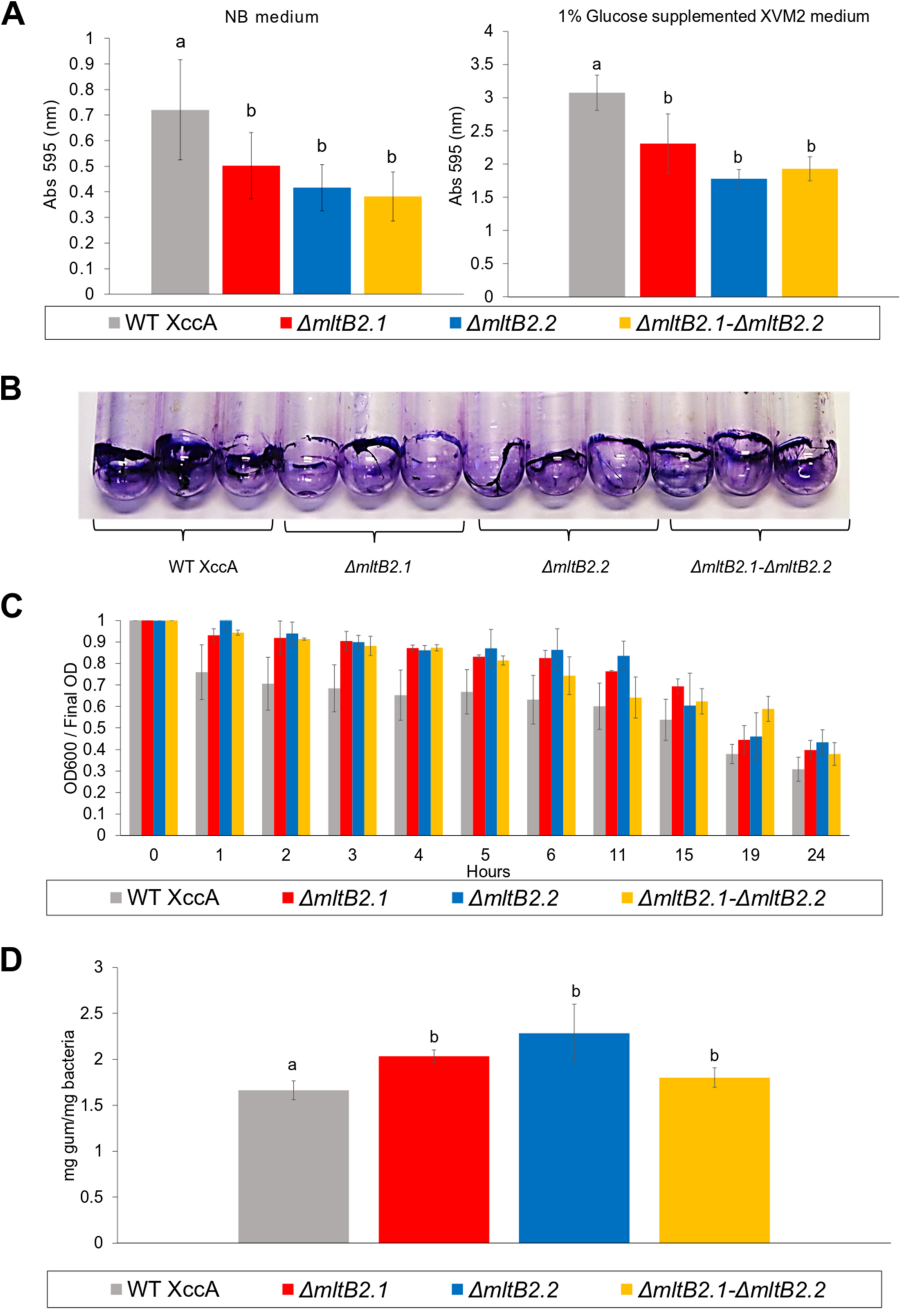
Biofilm formation, Cell Aggregation assay, and Xanthan gum quantification. (A) Biofilm formation assay. Measurement of biofilm formation by WT XccA and the mutants Δ*mltB2.1*, *ΔmltB2.2* and *ΔmltB2.1*–*ΔmltB2._2*. Assay performed using NB broth in static borosilicate glass tubes, and XVM2 defined media supplemented with 1% glucose (w/v) in static borosilicate glass tubes. Each bar is the mean of four independent experiments in which each strain was evaluated in quadruplicate. Error bars indicate the standard error. The data were statistically analyzed using *Tukey*’s test at 1% probability (*P* < 0.01). “a” and “b” indicate significant statistical difference among samples. (B) Stained biofilm for all strains on borosilicate glass tubes after assay performed using NB broth (biofilm formation for all strains on Microtitter plates and under the same conditions is shown in Fig. S4). (C) Cell Aggregation assay. Bars represent optical density readings at 600 nm in each strain over the course of 12 hours. Lower readings represent higher cell aggregation. Each bar is the mean of four independent experiments in which each strain was evaluated in quadruplicate. Error bars indicate the standard error. (D) Xanthan gum quantification. Extraction and quantification of xanthan gum on WT XccA and mutants *ΔmltB2.1*, *ΔmltB2._2* and *ΔmltB2._1*–*ΔmltB2.2*. Production increase is depicted in mg of gum produced by mg of bacteria extracted. Each bar is the mean of three independent experiments in which each strain was evaluated in triplicate. Error bars indicate the standard error. The data were statistically analyzed using Tukey’s test at 1% probability (*P* < 0.01). “a” and “b” indicate significant statistical difference among samples.

Biofilm formation, cell aggregation, xanthan gum production and bacterial motility are important features directly implied with XccA virulence. However, only slight differences in biofilm production between WT XccA and the mutants, *ΔmltB2*.1, *ΔmltB2*.2 and *ΔmltB2*.1–*ΔmltB2*.2, in both NB and in XVM2 media (which mimics the *in planta* conditions) were observed. There was no significant difference between the mutants and less than a twofold difference with the WT strain in NB medium (Fig. 4A), a difference which is further reduced in Glucose supplemented XVM2 medium (Fig. 4A). The differences in biofilm production between the mutants and WT XccA are more prominent when measured in borosilicate tubes and microtitter plates (Fig. 4B and Fig. S4), supporting a role for MltB2 in biofilm formation.

Since lower cell aggregation can lead to a reduction in biofilm production (Rigano et al., 2007), we tested the capacity of mutants and WT strains to aggregate (Materials and Methods), and to determine whether they correlate with the biofilm production assays. In spite of the results presented in the Fig. 4C which suggested that the WT strains have a slightly higher capacity to aggregate than the mutants, no statistical significance was observed, and therefore the lower biofilm production would not be directly related to cell aggregation (Fig. 4C).

Moreover, the measurement of xanthan gum production, which can also have an effect on the ability to form biofilms and to aggregate, also indicated only small differences between the mutant and WT strains (Fig. 4D).

**Figure 5:**
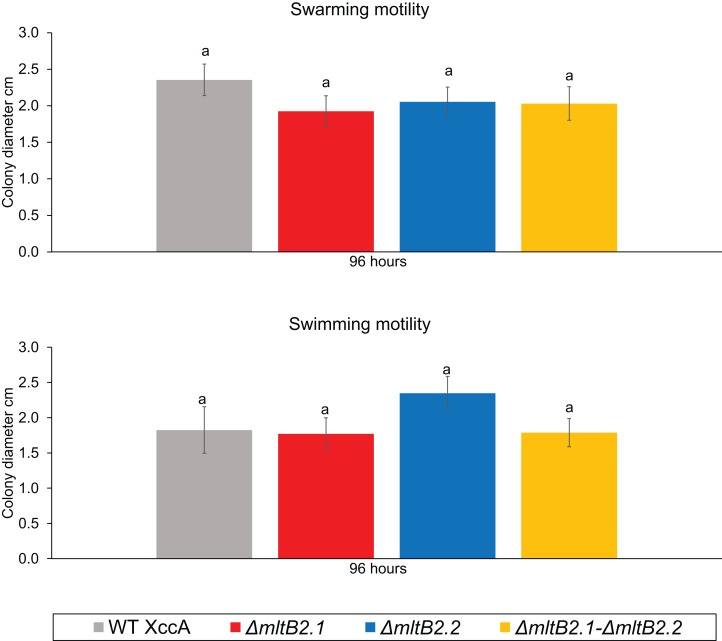
Bacterial Motility. *Swimming* motility (0.3% agar) of WT XccA and mutants *ΔmltB2.1*, *ΔmltB2.2* and *ΔmltB2.1*–*ΔmltB.2_.2*, and *swarming* motility (0.7% agar) of WT XccA and mutants *ΔmltB2.1*, *ΔmltB2.2* and *ΔmltB2.1*–*ΔmltB2._2*. Each bar is the mean of four independent experiments in which each strain was evaluated in quadruplicate. Error bars indicate the standard error. The data were statistically analyzed using Tukey’s test at 1% probability (*P* < 0.01). “a” indicates no significant statistical difference among samples.

Finally, since motility may also play a role in pathogenicity and virulence, we examined the potential effects on swarming and swimming behavior. Neither showed a significant difference between WT XccA and the three mutant configurations after 96 h (Fig. 5, and Fig. S5 for greater detail for 24, 48, 72 and 96 h), indicating that the Tn*Xax1* passenger LTs are not directly involved in flagellum assembly and bacterial motility.

Overall, these results indicate that the mutants *ΔmltB2.1*, *ΔmltB2.2*, and *ΔmltB2.1*–*ΔmltB2.2* do not affect cell aggregation or motility, but may play a small role in biofilm formation and xanthan gum production, thus supporting the idea of a link between MltB2 and XccA virulence mechanism.

## Discussion

### Lytic transglycosylase gene content of *Xanthomonas citri* pv *citri*

In view of the importance of bacterial LTs in general and the fact that we previously observed such genes associated with the Tn*3*-family transposon, Tn*Xax1*, identified in *Xanthomonas citri* pv citri (XccA), we initiated a study to determine the overall LT XccA gene content. We scanned its entire genome sequence (NC_003919, NC_003922 and NC_003921) using the LT gene list and classification according to Dik et al. (2017). This categorizes LTs into six families (one to six) and includes subfamilies (1A, 1B, 1C, 1D, 1E, 1F, 1G, 1H; 2A; 3A, 3B; 4A; 5A; 6A) (Dik et al., 2017). A total of 13 LTs were identified in XccA genome (Table 1 and Fig. S2), including the newly discovered *XAC_RS21660* LT, which is exclusively found in Xanthomonadales.

Interestingly, tblastn analysis against non-Xanthomonadales genomes revealed that *XAC_RS21660* hits are partitioned between two different and physically distant loci, mostly occurring in the *Pseudomonas* genus. Both loci form distinct ORFs: a homologue of *mltB2* LT from the 3B family and an epimerase related protein. The fact that these ORFs are located in different and distant loci in *Pseudomonas* suggests that *XAC_RS21660* may have been created by fusion of the two *Pseudomonas* ORFs. However, how this occurred remains to be established. This LT would also be a potential target for further studies since it is found exclusively in *Xanthomonadales*, and its role may also be related to that of the Tn*Xax1* 3B LT passenger genes, *mltB2*.1 and *mltB2*.2.

It is noteworthy that only a limited number of functional studies of LTs have been undertaken in *Xanthomonadales*: deletion of the 1G family member, *hpa2* (*PXO_RS00425*, 86% identity with *XAC_RS02185*), in *Xanthomonas oryzae* pv. *oryzae* (the causal agent of rice leaf bacterial blight) reduced the severity of disease symptoms and bacterial population on the leaf surface (Zhang et al., 2008), while the product of the *hpa2* gene (*XOC_RS19750*, 84% identity with *XAC_RS02185*) of *Xanthomonas oryzae* pv. *oryzicola* (the causative agent of leaf bacterial striatum, EBF, in rice) has been shown to interact with T3SS genes forming a complex to translocate effectors from the pathogen to the host (Li & Wang, 2011) and the *hpaH* homolog of *Xanthomonas campestris* pv. *vesicatoria* (*IS_RS03455*, 94% identity with *XAC_RS02145*) is located in the periplasm, where it binds to peptidoglycan and promotes the translocation of effectors into host cells (Hausner et al., 2017). These observations led to the belief that in XccA the 1G subfamily is also involved in insertion of type 3 and 4 secretion systems. Moreover, like the 1G family, proteins of the 1C subfamily are associated with cell wall remodeling and T4SS insertion (Santin & Cascales, 2017) as demonstrated for the *E. coli* MltE protein (Fibriansah, Gliubich & Thunnissen, 2012). There are two distinct members of the 1D subfamily and, although they include the LysM domain (IPR018392) (Fig. S2) commonly involved in plant-bacteria recognition (Spaink, 2004), their role in XccA has not been determined (Table 2). LTs belonging to families 1E, 1H, 2A and 4A were not identified in the XccA genome. Their absence is not surprising, since not all microorganisms would be expected to possess the entire known LT arsenal (Dik et al., 2017). Moreover, different LT families may show functional redundancy (Dik et al., 2017; Scheurwater, Reid & Clarke, 2008).

### Possible role(s) of Tn*Xax1*-associated *mltB2* genes in the XccA life-style

The occurrence of an *mltB2* copy in Tn*Xax1* both in pXAC64 and the XccA chromosome implies that this gene provides a selective advantage on its host. We have investigated various properties of XccA in which one or other or both *mltB* copies had been deleted.

The effect of the Tn*Xax1 mltB2*.1 and *mltB2*.2 passenger LT genes on XccA virulence and pathogenicity were revealed by inoculation using the spray method and by the more aggressive “inoculation” procedure (Figs. 1 and 2). Even though less dramatic than the results from the spray test, results from the inoculation assay showed that mutants *ΔmltB2*.1 and *ΔmltB2*.1–*ΔmltB2*.2 exhibit a clear delay in CC symptom development, evidenced by a reduction in hyperplastic tissue formation and complete absence of necrosis on later days compared to the wild strain (XccA WT). On the other hand, the chromosomal *ΔmltB2*.2 mutant showed very similar symptoms to those observed with WT XccA (no alterations in water soaking, hyperplastic tissue formation or necrosis) (Figs. 1 and 2).

The differential effect of the two *mltB2* mutations (shown in Figs. 1 and 2) might be the result of factors such as: a difference in expression of the two genes, a reduction in the activity of the chromosomal gene resulting from the single amino acid difference, or a difference in gene copy number between the plasmid and chromosomal copies. It seems unlikely that differential expression is responsible since the genes have an identical sequence environment within the Tn*Xax1* transposons. In particular, the putative promoter region (including the −35 and −10 region located upstream of the start codon) of *mltB2*.1 and *mltB2*.2 are identical. It also seems unlikely that the single amino acid difference is responsible since this occurs distant from the peptidoglycan binding-like and transglycosylase SLT 2 domains although the synonymous mutations could have an effect on expression at the translational level or on mRNA stability.

To determine whether the differences might be explained by copy number differences between the plasmid and chromosomal *mltB2* copies, we estimated these directly from data deposited in the NCBI SRA. Indeed, coverage ratio analysis indicated three pXAC64 copies per chromosomal origin of replication (Material and Methods). This is similar to the estimated copy number of pXOCgx01 and pXAP41 from *Xanthomonas oryzae* pv. *oryzicola* and *Xanthomonas arboricola* pv. *pruni*, four copies per cell (Niu et al., 2015; Pothier et al., 2011). Like pXAC64, those include conjugal transfer genes, Tn*Xax1* derivative elements and several virulence and pathogenicity genes, such as: heavy metal tolerance genes and T3SS effectors (Niu et al., 2015; Pothier et al., 2011).

The higher *mltB2*.1 plasmid-based copy number may therefore lead to an overall higher level of expression than the lower copy number chromosomal *mltB2*.2, explaining the observed results shown in Figs. 1 and 2.

Moreover, the ex vivo growth curve and plating tests on NB (defined medium) and SB (rich medium), suggests that MltB2 affects cell plating sensitivity or cell size (Figs. 3A and 3B). However, the behavior under other physiological conditions, such as survival in media with lower nutrient concentration than in NB, and/or an increasing level of production of potentially toxic compounds produced by the bacteria in both NB and SB media, warrants confirmation.

Nonetheless, the single and double MltB2 mutants are all able to propagate *in planta* (Fig. 3C). These findings support the notion that both MltB2 copies are indeed associated with XccA virulence, affecting the CC progression.

To explore the potential impact of the MltB2 in XccA cell in more detail, we examined a number of virulence related properties: biofilm, xanthan gum, motility and bacterial aggregation. These are all important characteristics related to bacterial virulence, and play key roles in pathogen-host interactions (Rigano et al., 2007). Indeed, biofilm production is advantageous for the bacterium because it offers protection against environmental stresses, mechanisms of defense by the host and production of antimicrobial compounds, besides promoting the development of the disease (Malamud et al., 2011).

All mltB2 mutants were found to form slightly lower biofilm levels (Fig. 4A), and slightly higher xanthan gum production (Fig. 4D) compared to WT XccA. *Although it* was not possible to establish that *mltB2*.1/*mltB2*.2 play a direct role in these phenomena or whether the differences are significant, these observations do suggest that MltB2 may play a subtle role in cell physiology and may influence assembly of other proteins important for biofilm, xanthan gum formation and secretion, and consequently, together, ultimately impacting CC development. The results also suggest the hypothesis that the lower production of biofilm observed in the mutant may increase the planktonic lifestyle of XccA rather than the production of mature biofilm that renders a more virulent microorganism, and therefore corroborating pathogenicity results shown in Figs. 1 and 2. Additionally, it seems that both MltB2 are not involved with cell motility and aggregation. According to the classification presented in the Table 2, we believe that the LT involved with the bacterial motility might be *XAC_RS13315* (*sltF*) from family 1F.

Taken together, these results reveal that *mltB2*.1 and *mltB2*.2 are not essential for XccA survival or CC development *in planta* since CC is delayed but eventually occurs in the absence of both genes. However, since deletion of the plasmid borne *mltB2*.1 results in a significant delay in CC intensity and appearance, this suggests that this gene product enhances XccA pathogenicity. While the results presented here do not reveal a detailed molecular picture of the role of MltB2, it is clear that both genes improve the disease promoting properties of the XccA host, perhaps by subtly influencing a number of processes involved, and thus would provide a selective advantage for their host bacteria.

## Conclusion

The major virulence and pathogenicity factors in XccA include T3SS, Type 2 secretion system, formation of biofilm and xanthan gum, QS, TCS as well as a suite of transcription activator-like effectors (TALEs) and *Xanthomonas* outer proteins (Xops) which are secreted by the T3SS (Ference et al., 2018).

We had previously shown not only that a number of effectors genes were passengers associated with Tn*3*-family transposons but also that the majority of TALE genes we had identified also form part of Tn*3*-like transposons. Indeed, a large number of these were in the form of mobile insertion cassettes in which they are flanked by long Tn*3*-family terminal inverted repeats but lack any of the Tn*3*-family transposition enzymes and whose transposition must depend on the availability of a cognate transposase supplied from another related transposon.

The results presented here demonstrate that another Tn*3*-family associated passenger gene, *mltB2*, are directly related with XccA virulence and affects CC progression. In addition, the data show that a plasmid-base copy of the gene, *mltB2*.1, had a larger effect than deletion of the chromosomal copy, *mltB2*.2. This is possibly due to a copy number effect since the pXAC64 plasmid is present in several copies per cell compared to the chromosomal copy. In spite of the presumably relationship with the peptidoglycan metabolism, neither gene was essential for XccA survival or CC development. However, more detailed biochemical and genetic studies are required to determine the exact function of *mltB2*.

This study has provided new insights into the cellular role of the MltB2 LTs, and together with information concerning the TALE genes (Ferreira et al., 2015), underlines the importance of mobile genetic elements, both plasmids and transposons, in the pathogenicity of XccA and, by inference, other Xanthomonads.

## Supplemental Information

10.7717/peerj.6111/supp-1 Supplemental Information 1 Figure S1. Schematic representation of the generation of *XAC_RS16355* (*mltB2*.2) and *XAC_RS22275* (*mltB2*.1) ORF deletion mutants.

10.7717/peerj.6111/supp-2 Supplemental Information 2 Figure S2. LTs found on *Xanthomonas citri* subsp. *citri* 306 genome. Alignment against the reference LTs according to Dick et al. (2017) and domain architecture.

10.7717/peerj.6111/supp-3 Supplemental Information 3 Figure S3. 1% agarose gels showing deletion *ΔmltB2*.1*, ΔmltB2*.2 and *ΔmltB2*.1*-ΔmltB2*.2 mutants confirmation. (M) 1Kb *Fermentas* *DNA Ladder* marker. ***ΔmltB2.1*** **mutant:** (C1) wt *mltB2*.2 gene, 2,100 bp; (C2) wt *mltB2*.1 gene, 1,100 bp; (1) wt *mltB2*.2 gene, 2,100 bp; (2) *ΔmltB2*.1 mutant, 500 bp; ***ΔmltB2.*****2**** **mutant:** (C1) *mltB2*.2 *wt* gene, 2,100 bp*; (C2) mltB2*.1 wt gene, 1,100 bp*;* (3) *ΔmltB2*.2, 750 bp; (4) *mltB2*.1 wt gene, 1,100 bp; ***ΔmltB2.*****1*****-ΔmltB2.*****2 double mutant:** (C1) *mltB2*.2 wt gene, 2,100 bp; (C2) *mltB2*.1 wt gene, 1,100 bp*;* (5) *ΔmltB2*.2 mutant, 750 bp; (6) *ΔmltB2*.1, 500 bp..

10.7717/peerj.6111/supp-4 Supplemental Information 4 Figure S4. Biofilm formation assay on Microtiter plates. Depiction of biofilm formation by WT XccA and the mutants *ΔmltB2*.1*, ΔmltB2*.2 and *ΔmltB2*.1*-ΔmltB2*.2. Assay performed using NB broth on Microtitter plates in order to compare results with the same assay performed in borosilicate glass tubes, under the same conditions. Each row is representative of five independent biological replicates. Results did not differ among Microtitter plates or borosilicate glass tubes.

10.7717/peerj.6111/supp-5 Supplemental Information 5 Figure S5. Bacterial Motility assay plates. Bacterial motility assay plates photographed after 24, 48, 72 and 96 hours. Each plate contains the WT XccA and mutants *ΔmltB2*.1*, ΔmltB2*.2 and *ΔmltB2*.1*-ΔmltB.2*.2. *Swarming* motility plates (0.7% agar) shown on top row. *Swimming* motility plates (0.3% agar) shown on bottom row.

10.7717/peerj.6111/supp-6 Supplemental Information 6 Table S1. Bacterial strains and plasmids used in this work.

10.7717/peerj.6111/supp-7 Supplemental Information 7 Cell Aggregation raw data.

10.7717/peerj.6111/supp-8 Supplemental Information 8 Biofilm raw data.

10.7717/peerj.6111/supp-9 Supplemental Information 9 *In planta* growth curve raw data.

10.7717/peerj.6111/supp-10 Supplemental Information 10 *Ex planta* growth curve raw data.

10.7717/peerj.6111/supp-11 Supplemental Information 11 NB and SB medium raw data.

10.7717/peerj.6111/supp-12 Supplemental Information 12 Cell motility raw data.

10.7717/peerj.6111/supp-13 Supplemental Information 13 Xanthan gum raw data.

## Additional Information and Declarations

### Data Availability

The following information was supplied regarding data availability: The raw data is available as Supplementary Material.

## References

[ref-1] Altschul SF, Madden TL, Schaffer AA, Zhang J, Zhang Z, Miller W, Lipman DJ (1997). Gapped BLAST and PSI-BLAST: a new generation of protein database search programs. Nucleic Acids Research.

[ref-2] Da Silva ACR, Ferro JA, Reinach FC, Farah CS, Furlan LR, Quaggio RB, Monteiro-Vitorello CB, Van Sluys MA, Almeida NF, Alves LMC, Do Amaral AM, Bertolini MC, Camargo LE, Camarotte G, Cannavan F, Cardozo J, Chambergo F, Ciapina LP, Cicarelli RM, Coutinho LL, Cursino-Santos JR, El-Dorry H, Faria JB, Ferreira AJ, Ferreira RC, Ferro MI, Formighieri EF, Franco MC, Greggio CC, Gruber A, Katsuyama AM, Kishi LT, Leite RP, Lemos EG, Lemos MV, Locali EC, Machado MA, Madeira AM, Martinez-Rossi NM, Martins EC, Meidanis J, Menck CF, Miyaki CY, Moon DH, Moreira LM, Novo MT, Okura VK, Oliveira MC, Oliveira VR, Pereira HA, Rossi A, Sena JA, Silva C, De Souza RF, Spinola LA, Takita MA, Tamura RE, Teixeira EC, Tezza RI, Trindade Dos Santos M, Truffi D, Tsai SM, White FF, Setubal JC, Kitajima JP (2002). Comparison of the genomes of two *Xanthomonas* pathogens with differing host specificities. Nature.

[ref-3] Dik DA, Marous DR, Fisher JF, Mobashery S (2017). Lytic transglycosylases: concinnity in concision of the bacterial cell wall. Critical Reviews in Biochemistry and Molecular Biology.

[ref-4] Do Amaral AM, Toledo CP, Baptista JC, Machado MA (2005). Transformation of *Xanthomonas axonopodis* pv. *citri* by electroporation. Fitopatologia Brasileira.

[ref-5] Ference CM, Gochez AM, Behlau F, Wang N, Graham JH, Jones JB (2018). Recent advances in the understanding of *Xanthomonas citri* ssp. *citri* pathogenesis and citrus canker disease management. Molecular Plant Pathology.

[ref-6] Ferreira RM, De Oliveira AC, Moreira LM, Belasque J, Gourbeyre E, Siguier P, Ferro MI, Ferro JA, Chandler M, Varani AM (2015). A TALE of transposition: Tn3-like transposons play a major role in the spread of pathogenicity determinants of *Xanthomonas citri* and other xanthomonads. mBio.

[ref-7] Ferreira RM, Moreira LM, Ferro JA, Soares MRR, Laia ML, Varani AM, de Oliveira JCF, Ferro MIT (2016). Unravelling potential virulence factor candidates in *Xanthomonas citri*. subsp. *citri* by secretome analysis. PeerJ.

[ref-8] Fibriansah G, Gliubich FI, Thunnissen AM (2012). On the mechanism of peptidoglycan binding and cleavage by the endo-specific lytic transglycosylase MltE from *Escherichia coli*. Biochemistry.

[ref-9] Finn RD, Attwood TK, Babbitt PC, Bateman A, Bork P, Bridge AJ, Chang HY, Dosztanyi Z, El-Gebali S, Fraser M, Gough J, Haft D, Holliday GL, Huang H, Huang X, Letunic I, Lopez R, Lu S, Marchler-Bauer A, Mi H, Mistry J, Natale DA, Necci M, Nuka G, Orengo CA, Park Y, Pesseat S, Piovesan D, Potter SC, Rawlings ND, Redaschi N, Richardson L, Rivoire C, Sangrador-Vegas A, Sigrist C, Sillitoe I, Smithers B, Squizzato S, Sutton G, Thanki N, Thomas PD, Tosatto SCE, Wu CH, Xenarios I, Yeh LS, Young SY, Mitchell AL (2017). InterPro in 2017–beyond protein family and domain annotations. Nucleic Acids Research.

[ref-10] Gochez AM, Huguet-Tapia JC, Minsavage GV, Shantaraj D, Jalan N, Strauss A, Lahaye T, Wang N, Canteros BI, Jones JB, Potnis N (2018). Pacbio sequencing of copper-tolerant *Xanthomonas citri* reveals presence of a chimeric plasmid structure and provides insights into reassortment and shuffling of transcription activator-like effectors among *X. citri* strains. BMC Genomics.

[ref-11] Graham JH, Gottwald TR, Cubero J, Achor DS (2004). *Xanthomonas axonopodis* pv. *citri*: factors affecting successful eradication of citrus canker. Molecular Plant Pathology.

[ref-12] Granato LM, Picchi SC, Andrade MdeO, Takita MA, De Souza AA, Wang N, Machado MA (2016). The ATP-dependent RNA helicase HrpB plays an important role in motility and biofilm formation in *Xanthomonas citri* subsp. *citri*. BMC Microbiology.

[ref-13] Hausner J, Hartmann N, Jordan M, Buttner D (2017). The predicted lytic transglycosylase HpaH from *Xanthomonas campestris* pv. *vesicatoria* associates with the Type III secretion system and promotes effector protein translocation. Infection Immunology.

[ref-14] Holtje JV (1998). Growth of the stress-bearing and shape-maintaining murein sacculus of *Escherichia coli*. Microbiology and Molecular Biology Reviews.

[ref-15] Jalan N, Kumar D, Andrade MO, Yu F, Jones JB, Graham JH, White FF, Setubal JC, Wang N (2013). Comparative genomic and transcriptome analyses of pathotypes of *Xanthomonas citri* subsp. *citri* provide insights into mechanisms of bacterial virulence and host range. BMC Genomics.

[ref-16] Jalan NY, Kogenaru QS, Guo Y, Jones JB, Graham J, Wang Y, Gross DC, Lichens-Park A, Kole C (2014). Genomics of Xanthomonas citri and related species. Genomic of Plant-associated Bacteria.

[ref-17] Kaniga K, Delor I, Cornelis GR (1991). A wide-host-range suicide vector for improving reverse genetics in Gram-negative bacteria: inactivation of the *blaA* gene of *Yersinia enterocolitica*. Gene.

[ref-18] Lacerda LA, Cavalca LB, Martins PMM, Govone JS, Bacci M, Ferreira H (2017). Protein depletion using the arabinose promoter in *Xanthomonas citri* subsp. *citri*. Plasmid.

[ref-19] Laia ML, Moreira LM, Dezajacomo J, Brigati JB, Ferreira CB, Ferro MI, Silva ACR, Ferro JA, Oliveira JC (2009). New genes of *Xanthomonas citri* subsp. *citri* involved in pathogenesis and adaptation revealed by a transposon-based mutant library. BMC Microbiology.

[ref-20] Langmead B, Salzberg SL (2012). Fast gapped-read alignment with Bowtie 2. Nature Methods.

[ref-21] Larkin MA, Blackshields G, Brown NP, Chenna R, McGettigan PA, McWilliam H, Valentin F, Wallace IM, Wilm A, Lopez R, Thompson JD, Gibson TJ, Higgins DG (2007). Clustal W and Clustal X version 2.0. Bioinformatics.

[ref-39] Lee J, Lee HJ, Shin MK, Ryu WS (2004). Versatile PCR-mediated insertion or deletion mutagenesis. Biotechniques.

[ref-22] Li H, Handsaker B, Wysoker A, Fennell T, Ruan J, Homer N, Marth G, Abecasis G, Durbin R, 1000 Genome Project Data Processing Subgroup (2009). The sequence alignment/map format and SAMtools. Bioinformatics.

[ref-23] Li J, Wang N (2011). The *wxacO* gene of *Xanthomonas citri* sp. *citri* encodes a protein with a role in lipopolysaccharide biosynthesis, biofilm formation, stress tolerance and virulence. Molecular Plant Patholology.

[ref-24] Malamud F, Torres PS, Roeschlin R, Rigano LA, Enrique R, Bonomi HR, Castagnaro AP, Marano MR, Vojnov AA (2011). The *Xanthomonas axonopodis* pv. *citri* flagellum is required for mature biofilm and canker development. Microbiology.

[ref-25] Mansfield J, Genin S, Magori S, Citovsky V, Sriariyanum M, Ronald P, Dow M, Verdier V, Beer SV, Machado MA, Toth I, Salmond G, Foster GD (2012). Top 10 plant pathogenic bacteria in molecular plant pathology. Molecular Plant Pathology.

[ref-26] Nicolas E, Lambin M, Dandoy D, Galloy C, Nguyen N, Oger CA, Hallet B (2015). The Tn3-family of replicative transposons. Microbiology Spectrum.

[ref-27] Niu XN, Wei ZQ, Zou HF, Xie GG, Wu F, Li KJ, Jiang W, Tang JL, He YQ (2015). Complete sequence and detailed analysis of the first indigenous plasmid from *Xanthomonas oryzae* pv. *oryzicola*. BMC Microbiology.

[ref-28] Pothier JF, Vorholter FJ, Blom J, Goesmann A, Puhler A, Smits THM, Duffy B (2011). The ubiquitous plasmid pXap41 in the invasive phytopathogen *Xanthomonas arboricola* pv. *pruni*: complete sequence and comparative genomic analysis. FEMS Microbiology Letters.

[ref-29] Raetz CRH, Whitfield C (2002). Lipopolysaccharide endotoxins. Annual Review of Biochemistry.

[ref-30] Rigano LA, Siciliano F, Enrique R, Sendin L, Filippone P, Torres PS, Questa J, Dow JM, Castagnaro AP, Vojnov AA, Marano MR (2007). Biofilm formation, epiphytic fitness, and canker development in *Xanthomonas axonopodis* pv. *citri*. Molecular Plant-Microbe Interactions.

[ref-31] Sambrook J, Fritschi EF, Maniatis T (1989). Plasmid vector. Molecular Cloning: A Laboratory Manual.

[ref-32] Santin YG, Cascales E (2017). Domestication of a housekeeping transglycosylase for assembly of a Type VI secretion system. EMBO Reports.

[ref-33] Scheurwater E, Reid CW, Clarke AJ (2008). Lytic transglycosylases: bacterial space-making autolysins. International Journal of Biochemistry & Cell Biology.

[ref-34] Shu CH, Yang ST (1990). Effects of temperature on cell growth and xanthan production in batch cultures of *Xanthomonas campestris*. Biotechnology and Bioengineering.

[ref-35] Spaink HP (2004). Specific recognition of bacteria by plant LysM domain receptor kinases. Trends in Microbiololy.

[ref-36] Yan Q, Hu X, Wang N (2012). The novel virulence-related gene *nlxA* in the lipopolysaccharide cluster of *Xanthomonas citri ssp. citri* is involved in the production of lipopolysaccharide and extracellular polysaccharide, motility, biofilm formation and stress resistance. Molecular Plant Pathology.

[ref-37] Yan Q, Wang N (2011). The ColR/ColS two-component system plays multiple roles in the pathogenicity of the citrus canker pathogen *Xanthomonas citri* subsp. *citri*. Journal of Bacteriology.

[ref-38] Zhang J, Wang X, Zhang Y, Zhang G, Wang J (2008). A conserved Hpa2 protein has lytic activity against the bacterial cell wall in phytopathogenic *Xanthomonas oryzae*. Applied Environmental and Microbiology.

