# Supplementary figures and images for: Transposons and pathogenicity in Xanthomonas: acquisition of murein lytic transglycosylases by TnXax1 enhances Xanthomonas citri subsp. citri 306 virulence and fitness
Source: PeerJ. 2018 Dec 19;6:e6111. doi: 10.7717/peerj.6111 (PMC6304161; doi:10.7717/peerj.6111)

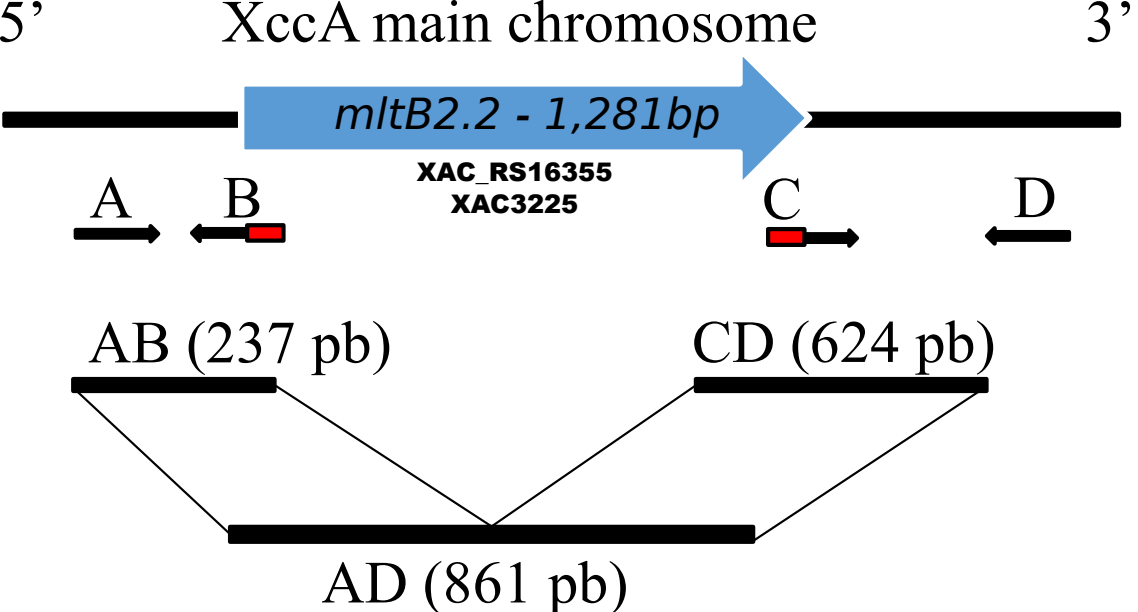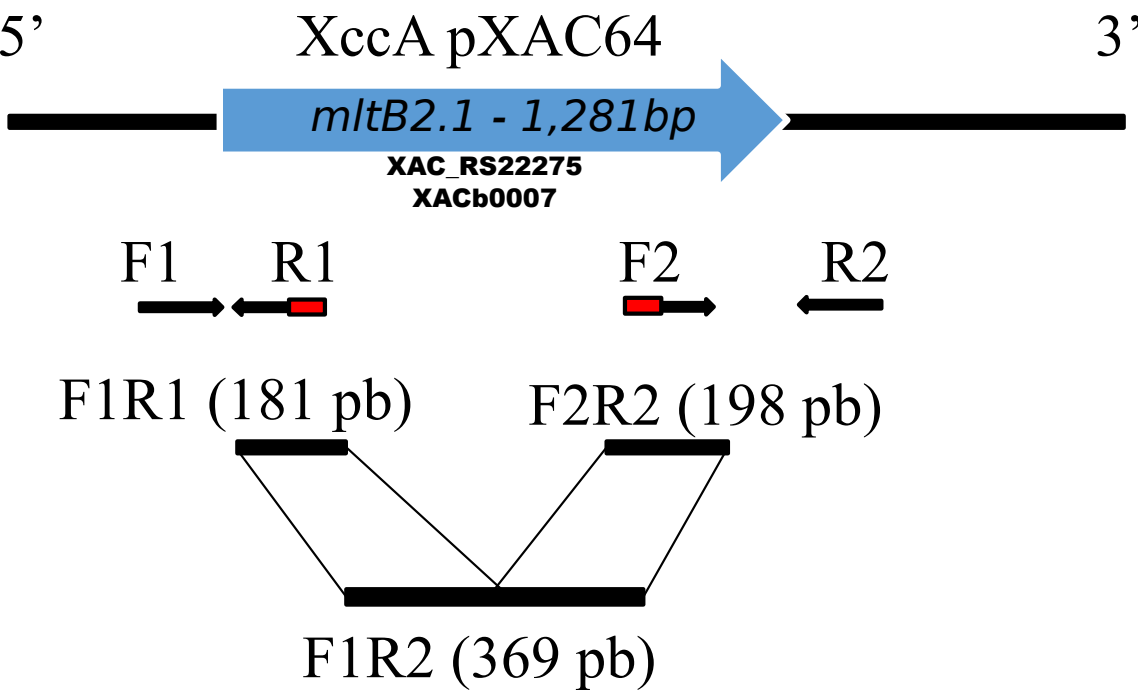

Supplement: Supplemental Information 1 [file peerj-06-6111-s001.pdf]

# Biological Replicates

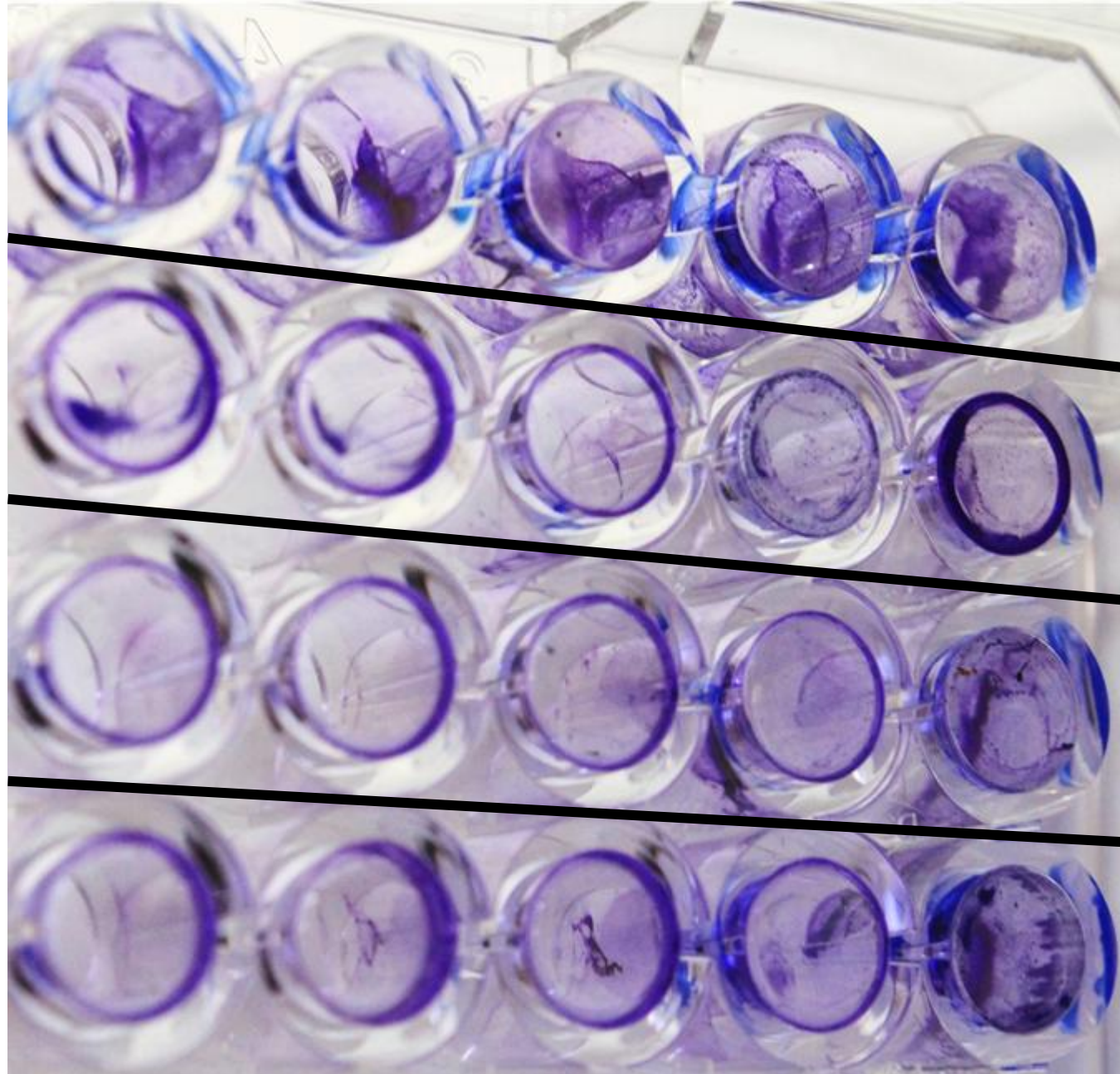

→ WT XccA

→  $\Delta mltB2.1$

→  $\Delta mltB2.2$

→  $\Delta mltB2.1-\Delta mltB2.2$

Supplement: Supplemental Information 4 — Depiction of biofilm formation by WT XccA and the mutants ΔmltB2.1, ΔmltB2.2 and ΔmltB2.1-ΔmltB2.2. Assay performed using NB broth on Microtitter plates in order to compare results with the same assay performed in borosilicate glass tubes, under the same conditions. Each row is representative of five independent biological replicates. Results did not differ among Microtitter plates or borosilicate glass tubes. [file peerj-06-6111-s004.pdf]

Swarming

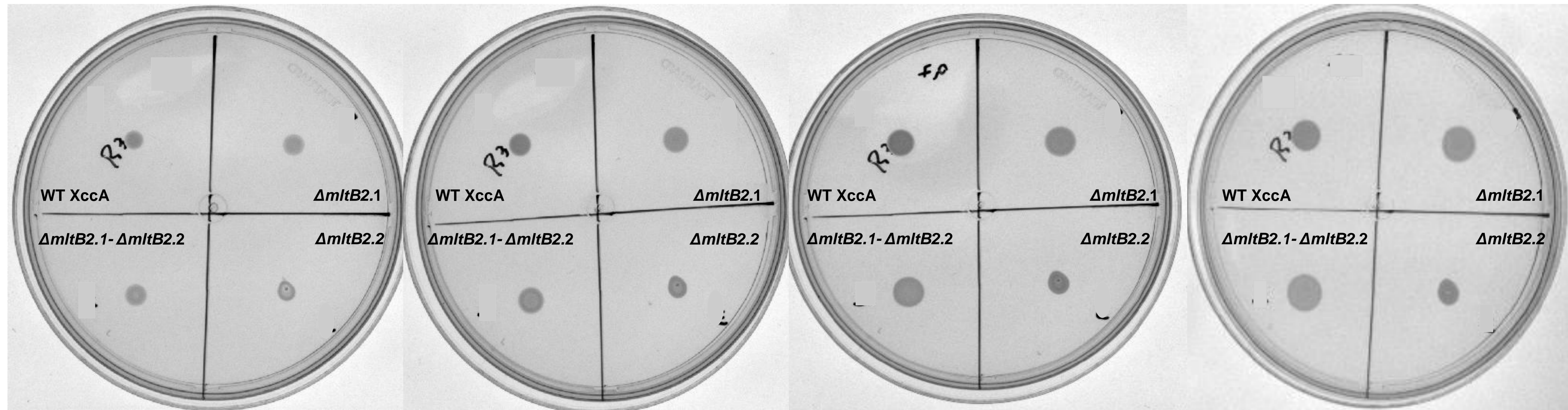

24 hours

48 hours

72 hours

96 hours

Swimming

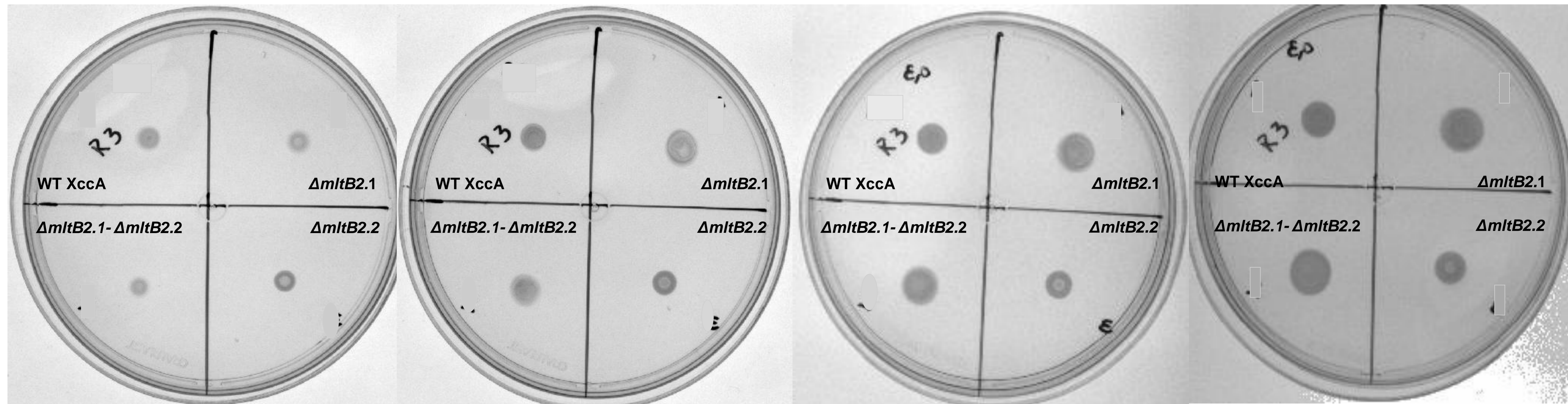

Supplement: Supplemental Information 5 — Bacterial motility assay plates photographed after 24, 48, 72 and 96 hours. Each plate contains the WT XccA and mutants ΔmltB2.1, ΔmltB2.2 and ΔmltB2.1-ΔmltB.2.2. Swarming motility plates (0.7% agar) shown on top row. Swimming motility plates (0.3% agar) shown on bottom row. [file peerj-06-6111-s005.pdf]
