# Supplementary material for: Transposons and pathogenicity in Xanthomonas: acquisition of murein lytic transglycosylases by TnXax1 enhances Xanthomonas citri subsp. citri 306 virulence and fitness
Source: PeerJ. 2018 Dec 19;6:e6111. doi: 10.7717/peerj.6111 (PMC6304161; doi:10.7717/peerj.6111)
Supplement: Supplemental Information 2 — Alignment against the reference LTs according to Dick et al. (2017) and domain architecture. [file peerj-06-6111-s002.pdf]

Family 1A - Alignment

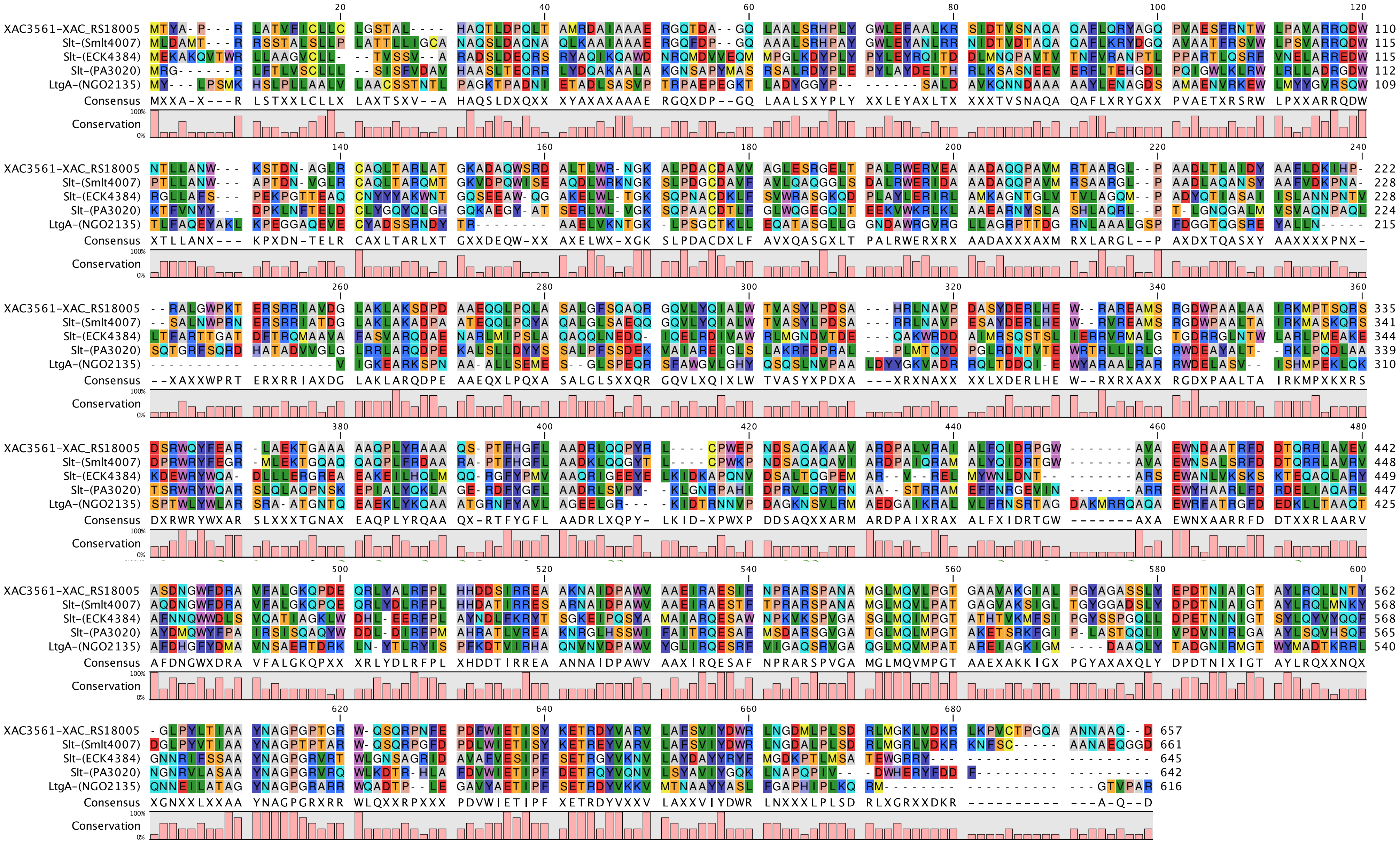

Family 1A - Domain architecture

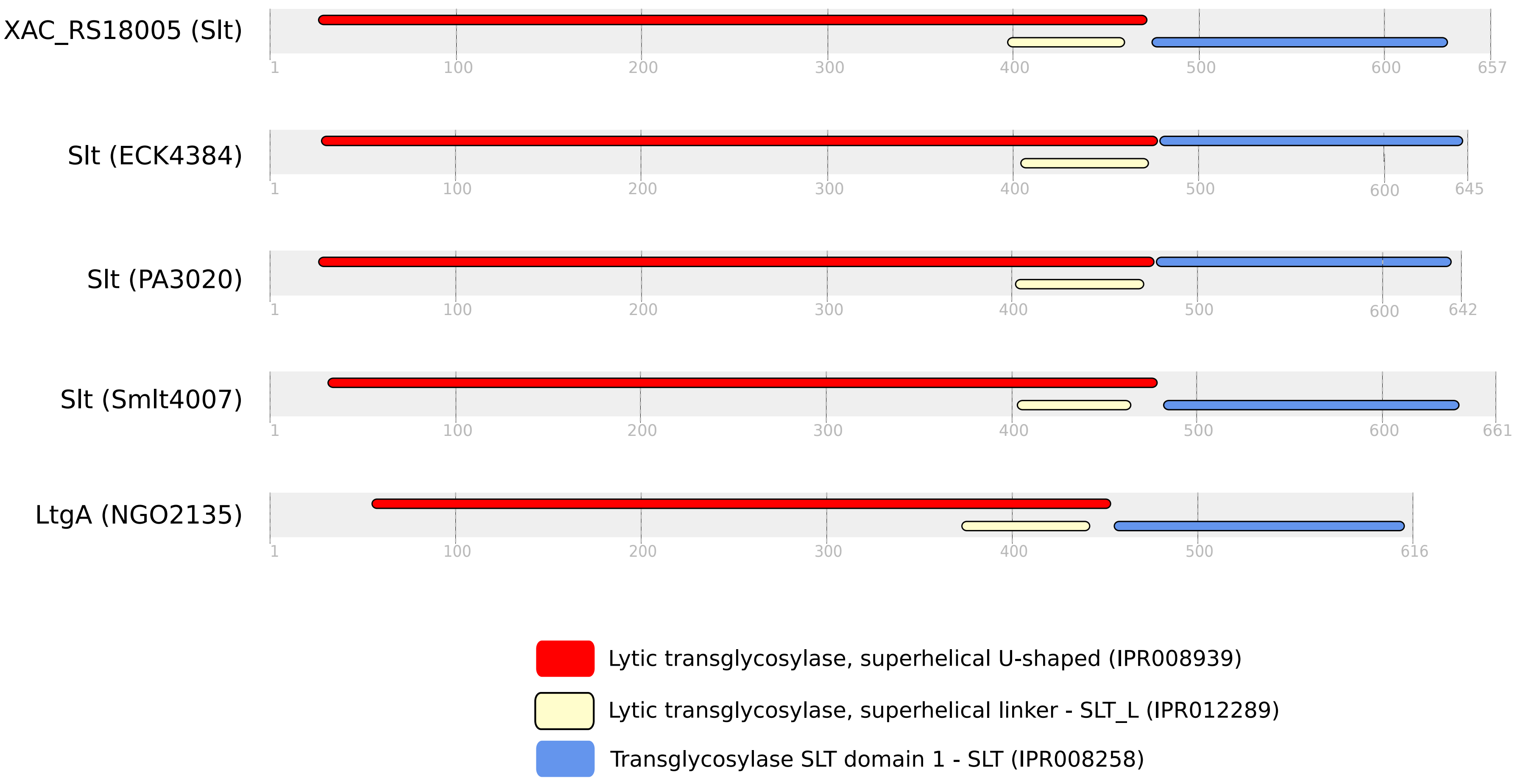

Family 1B - Alignment

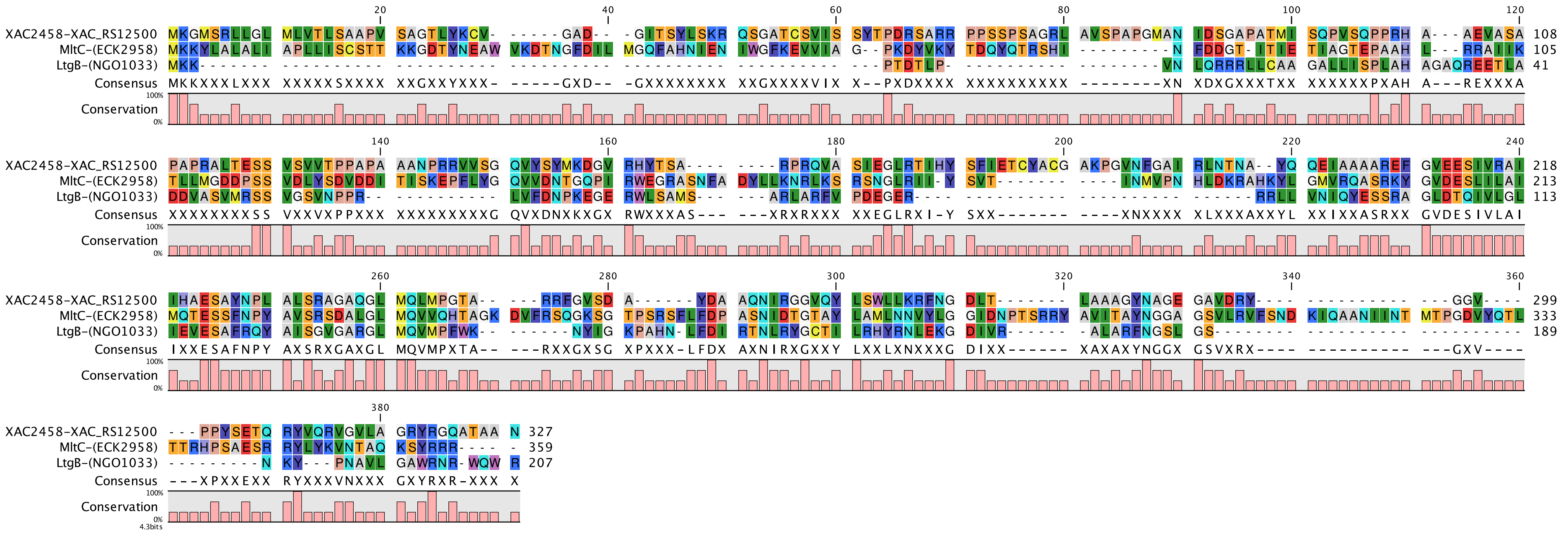

Family 1B - Domain architecture

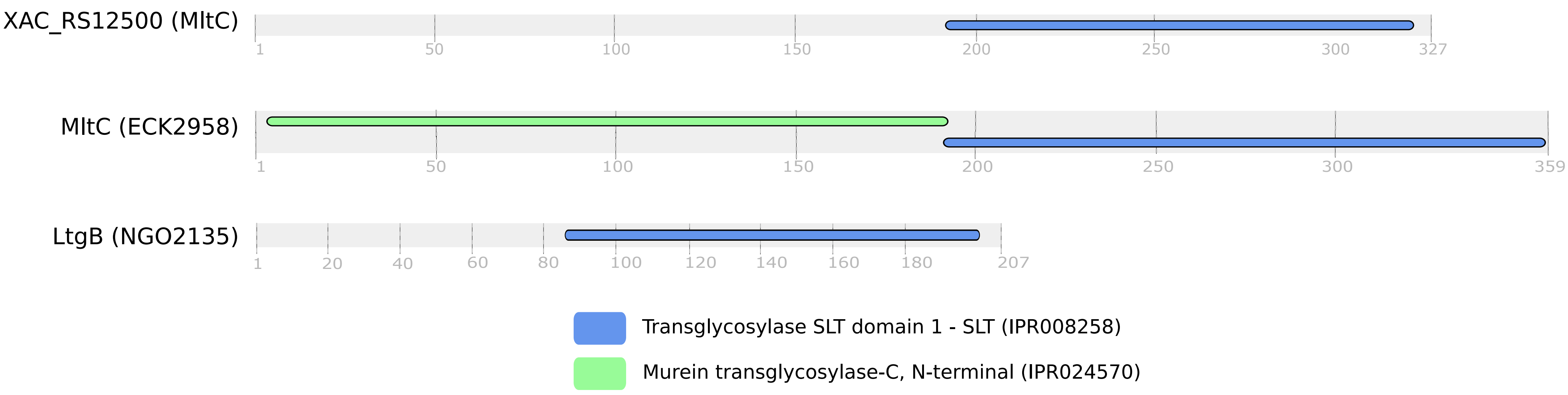

Family 1C - Alignment

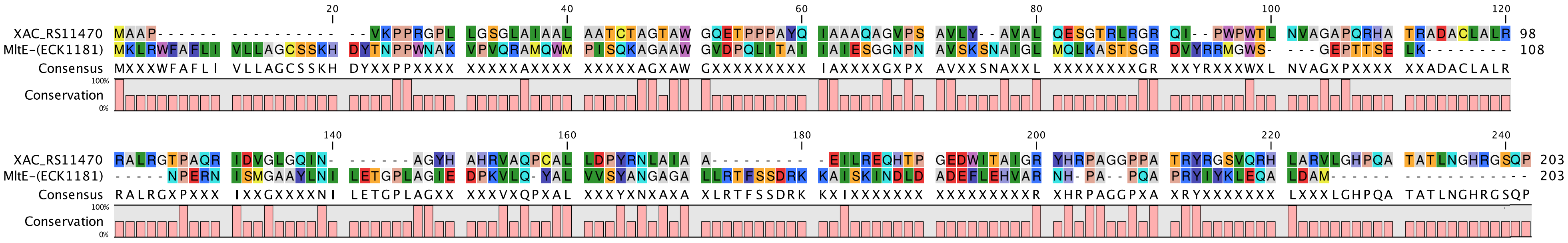

Family 1C - Domain architecture

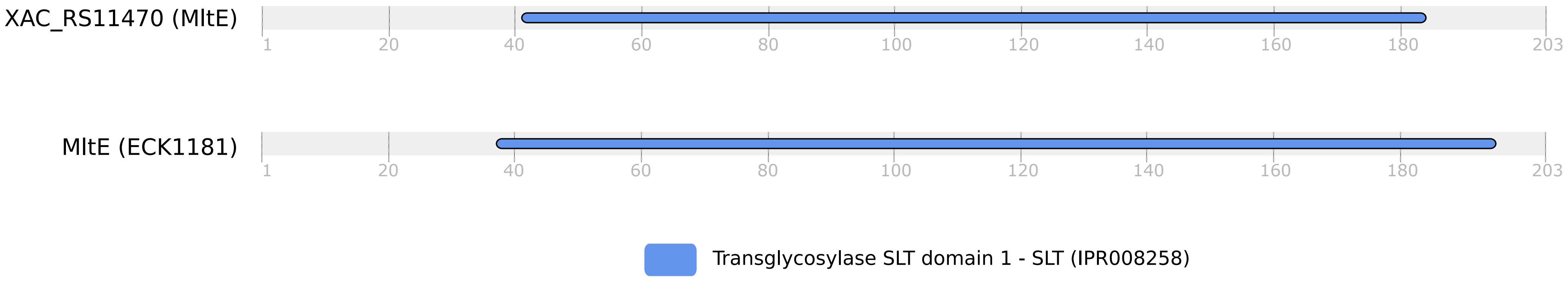

Family 1D - Alignment

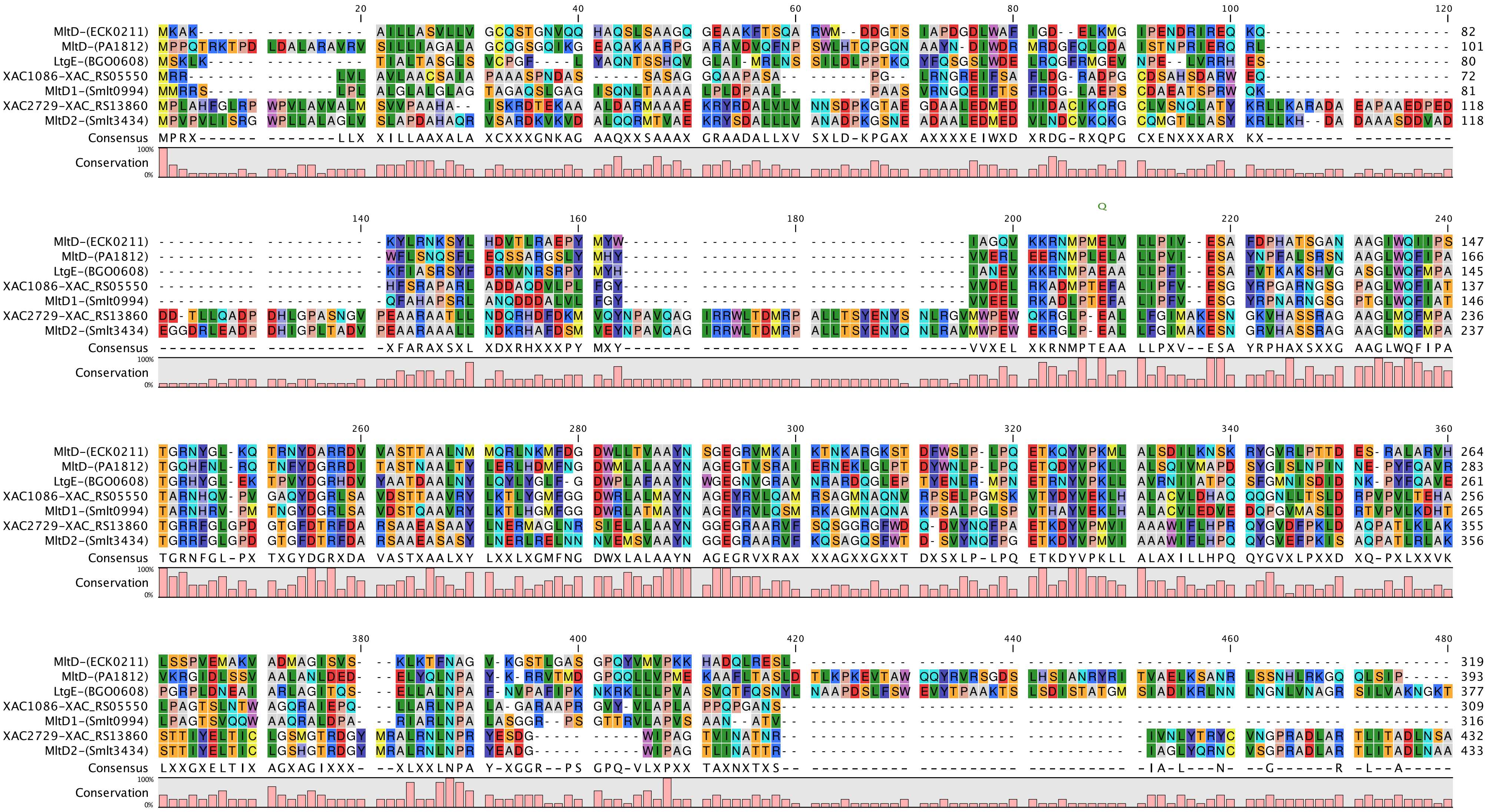

continue in the next page

Family 1D - Alignment

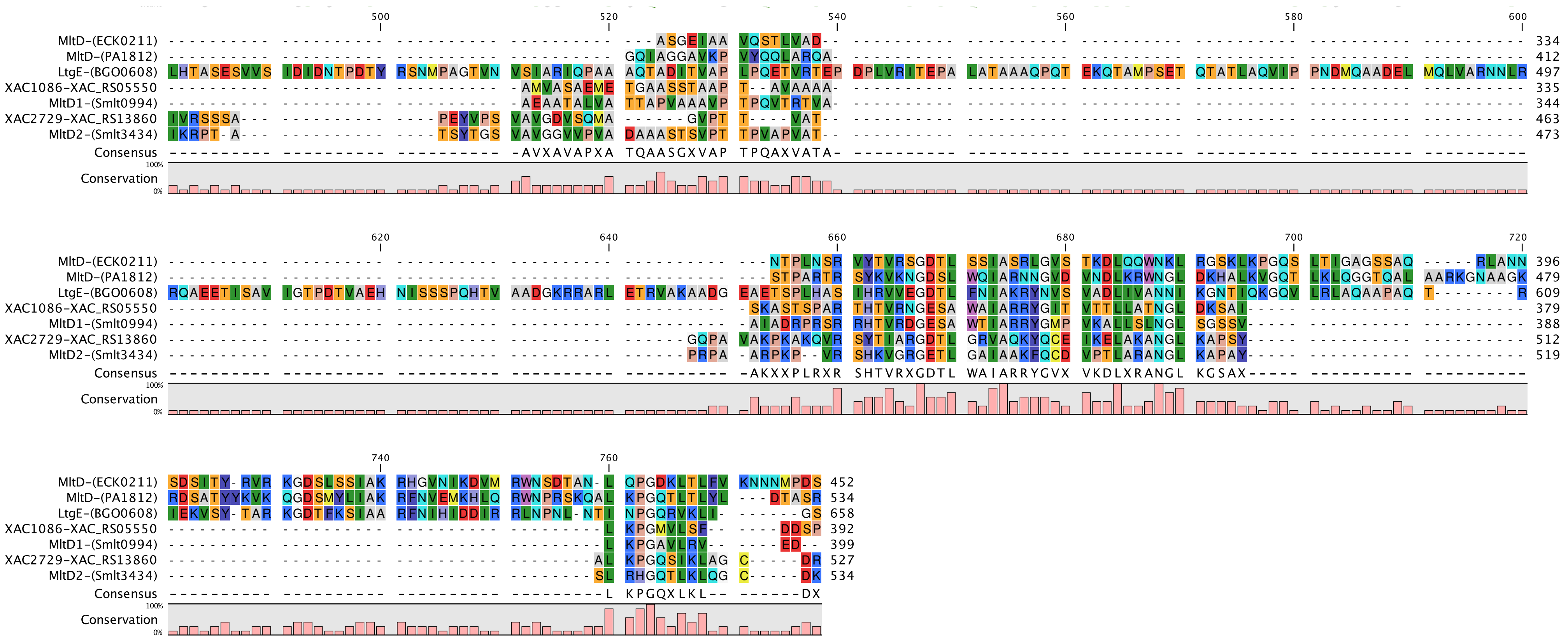

Family 1D - Domain architecture

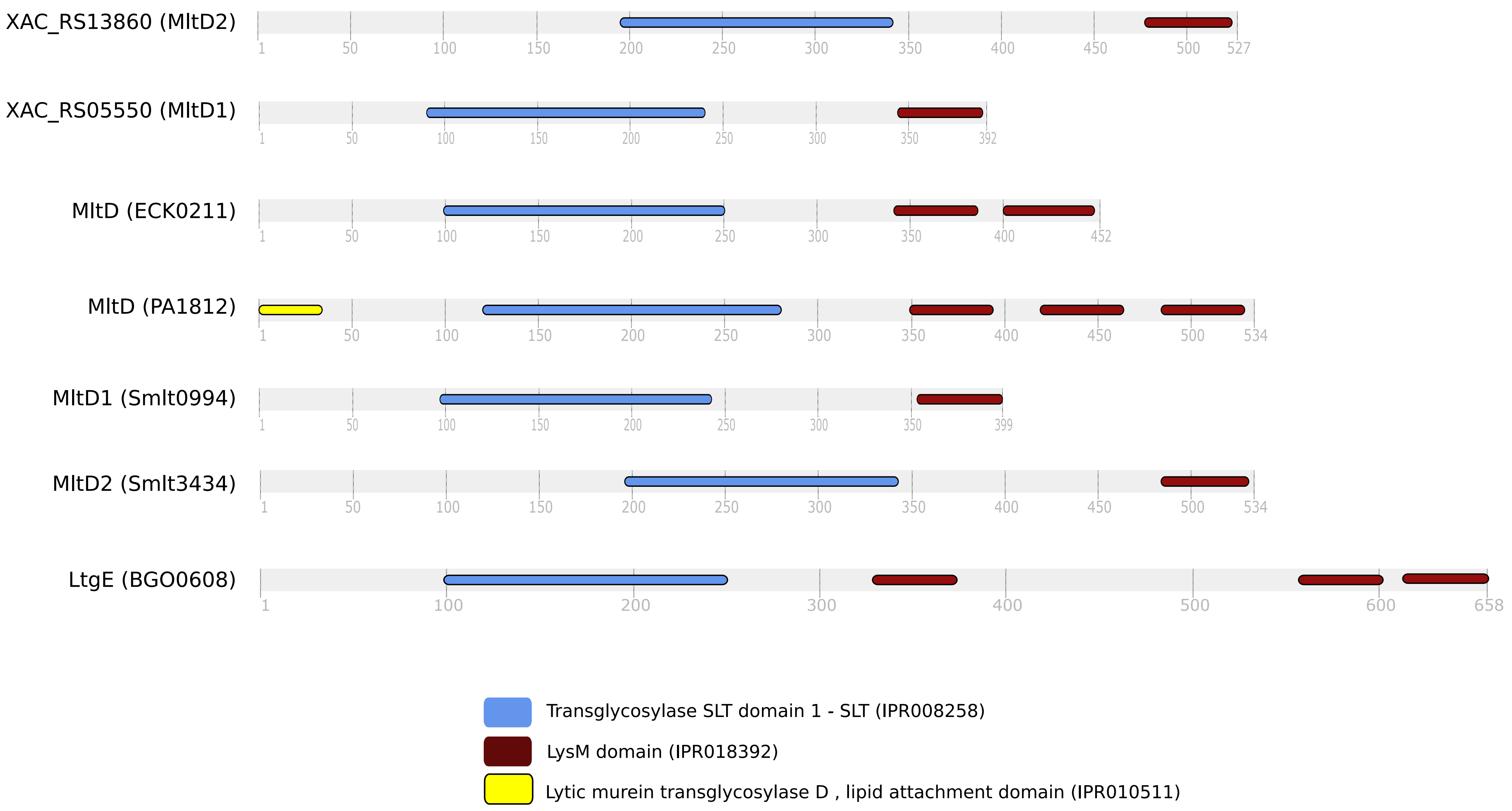

Family 1F - Alignment

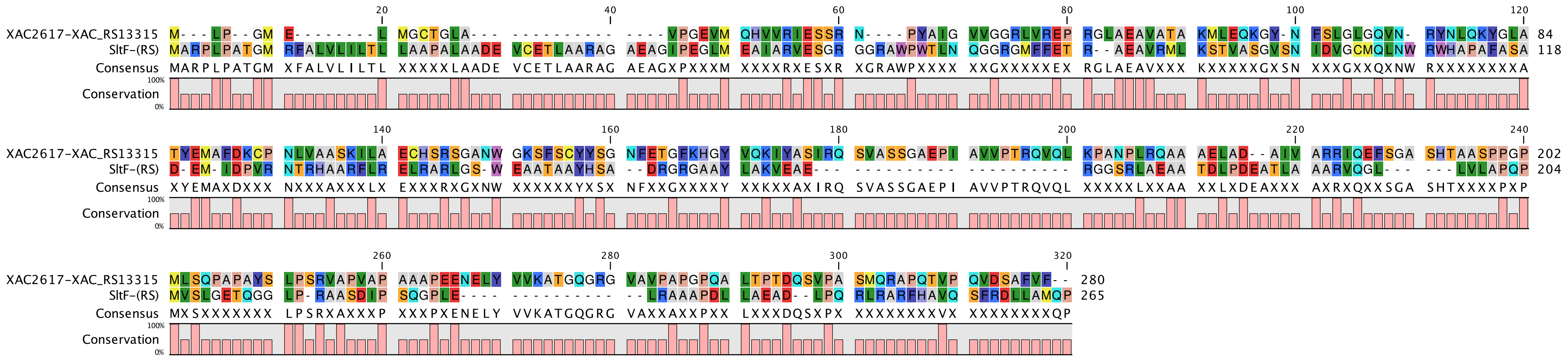

Family 1F - Domain architecture

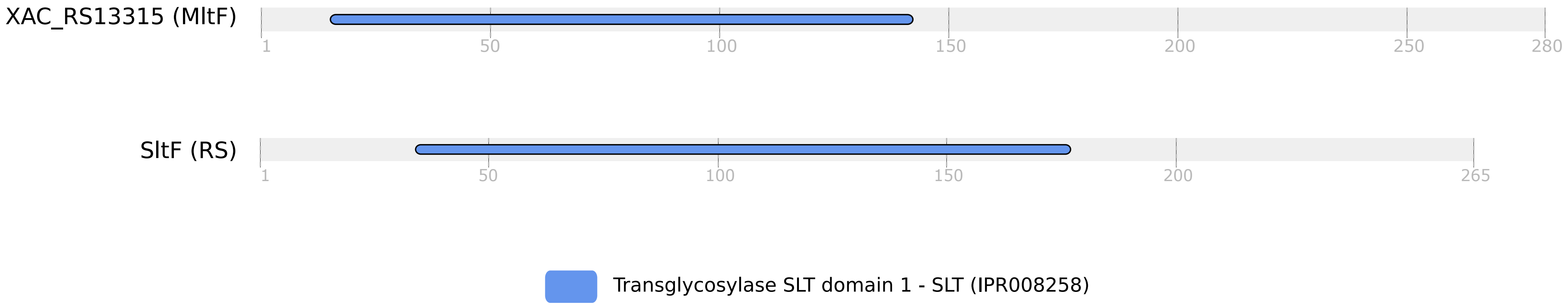

Family 1G - Alignment

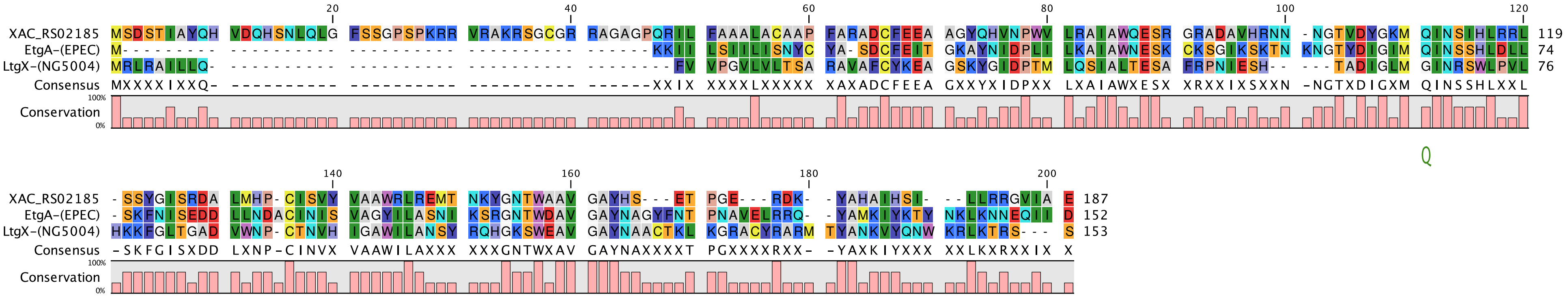

Family 1G - Domain architecture

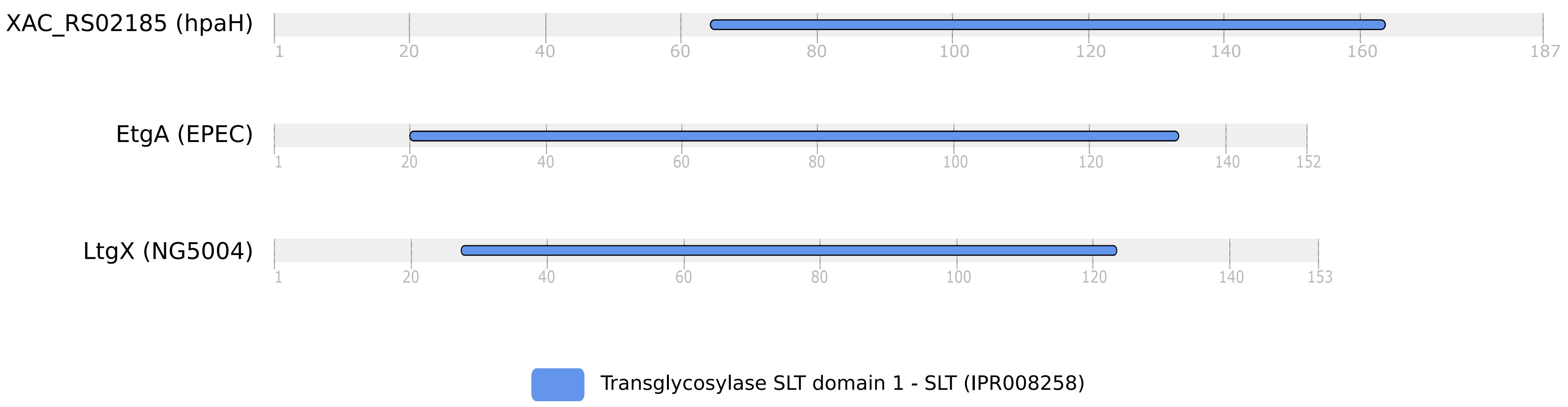

Family 3A - Alignment

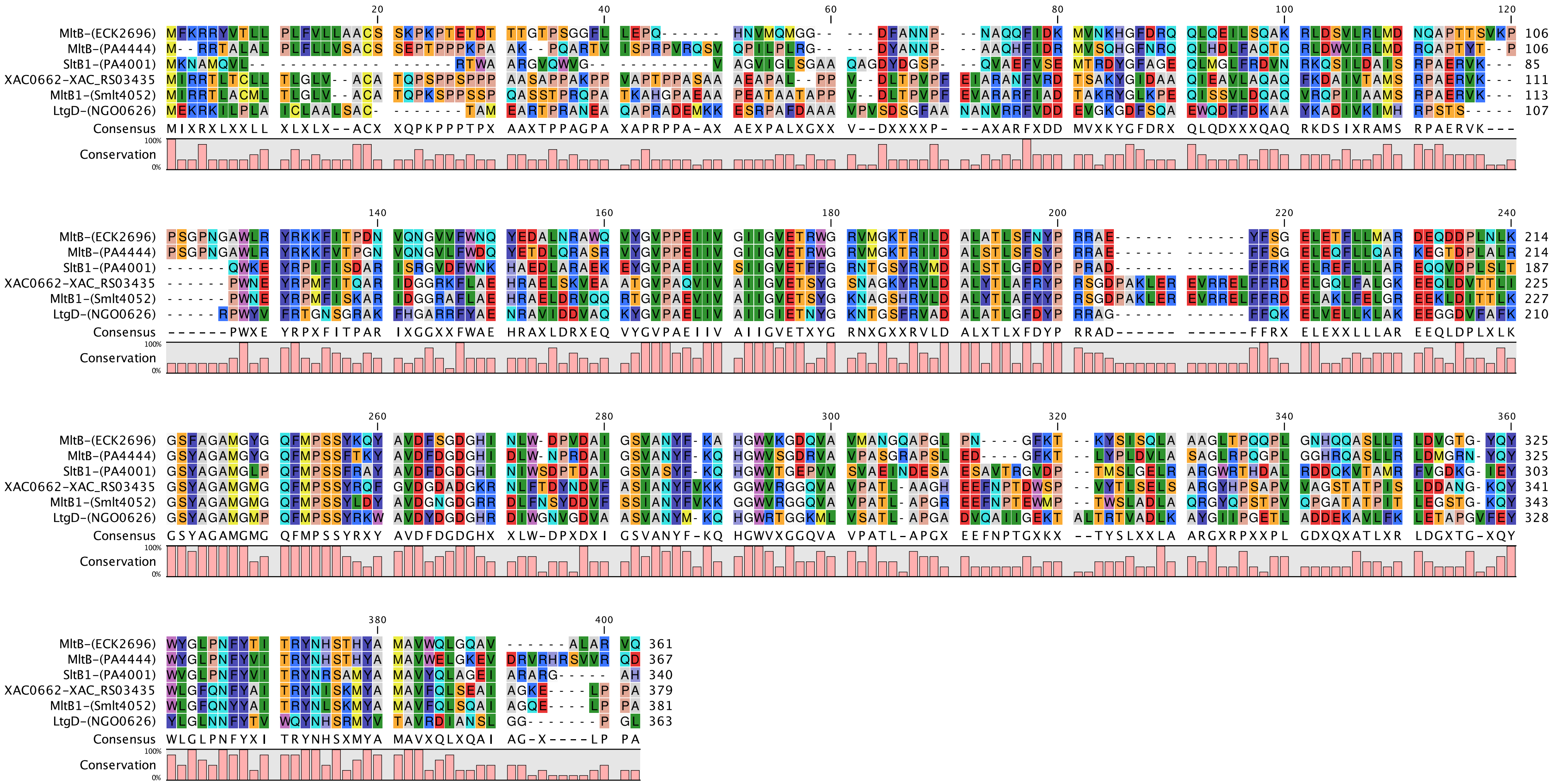

Family 3A - Domain architecture

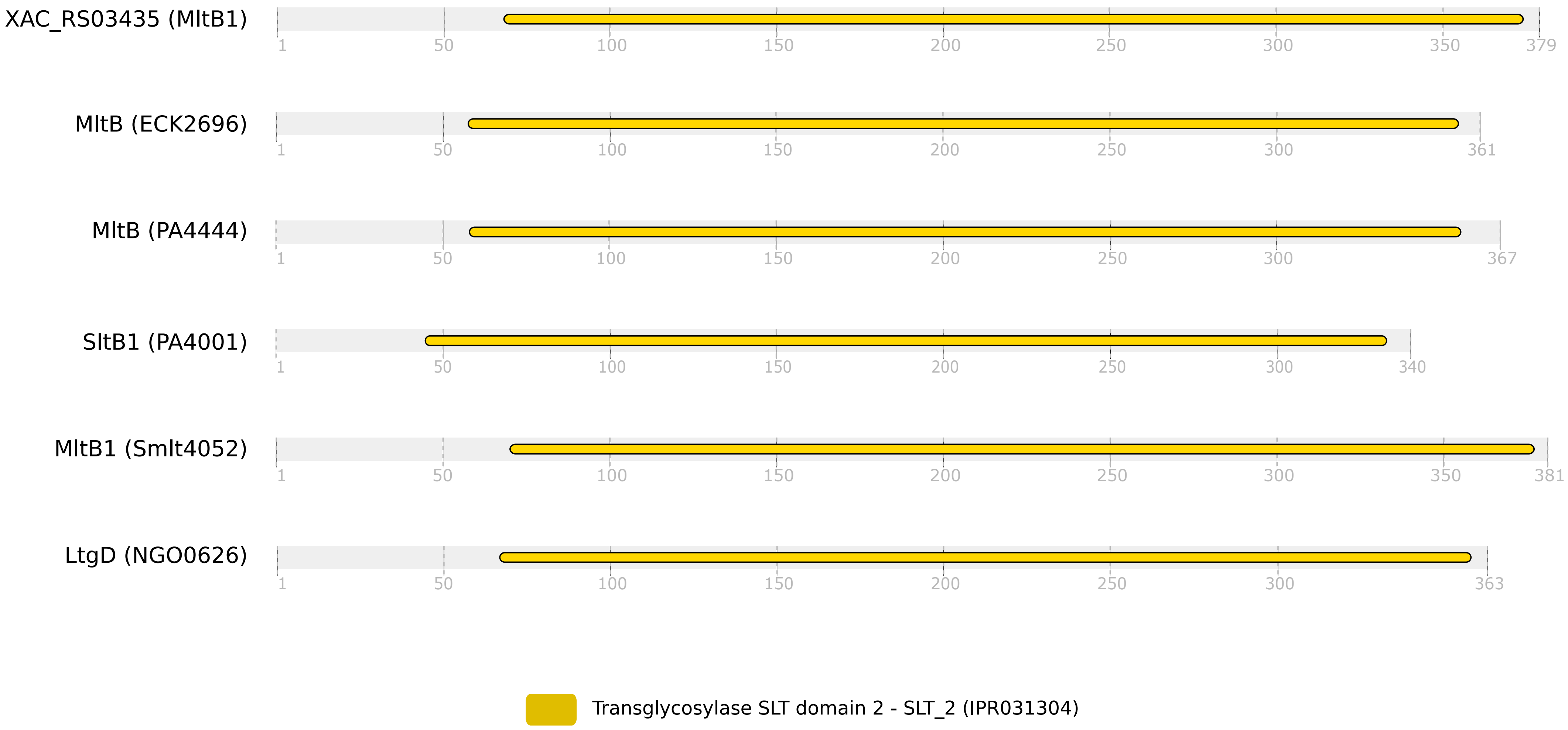

Family 3B - Alignment

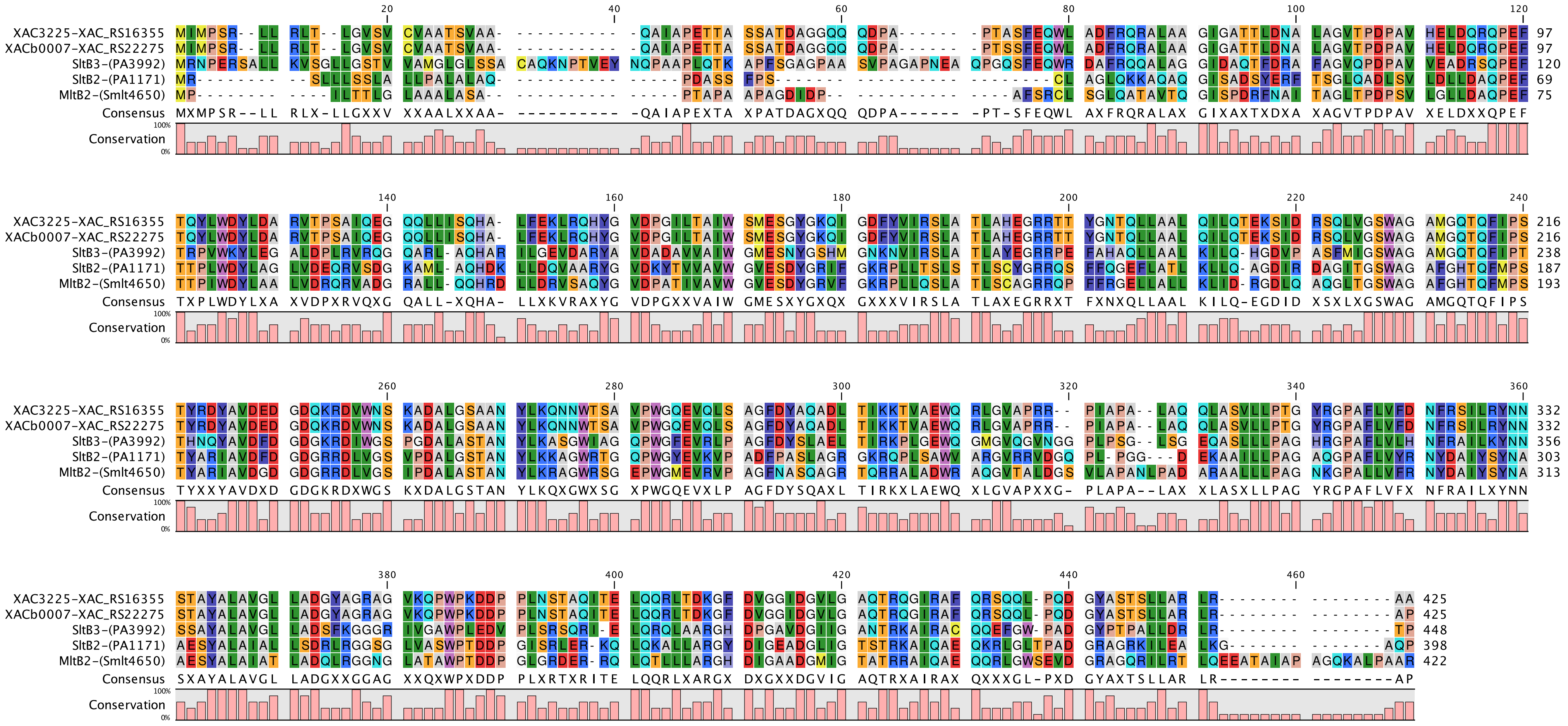

Family 3B - Domain architecture

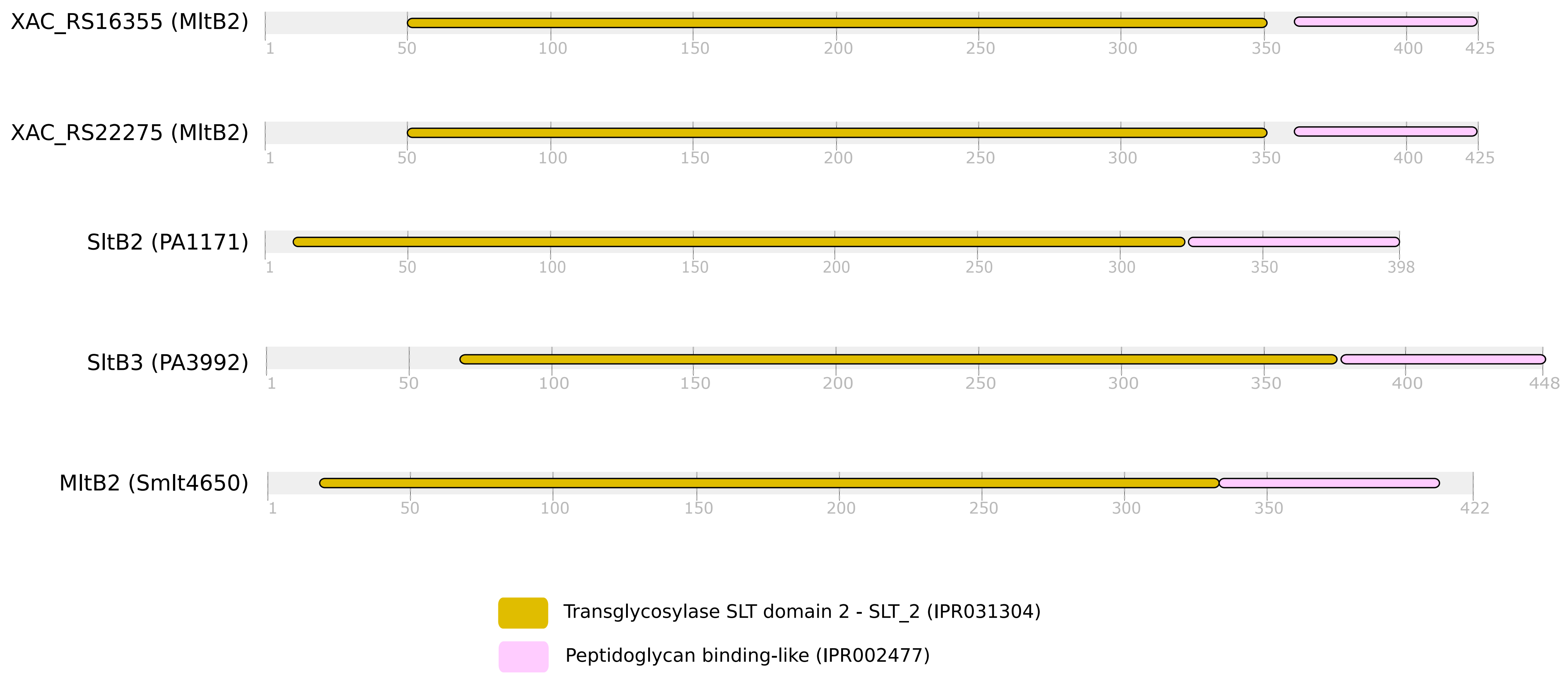

Family 5A - Alignment

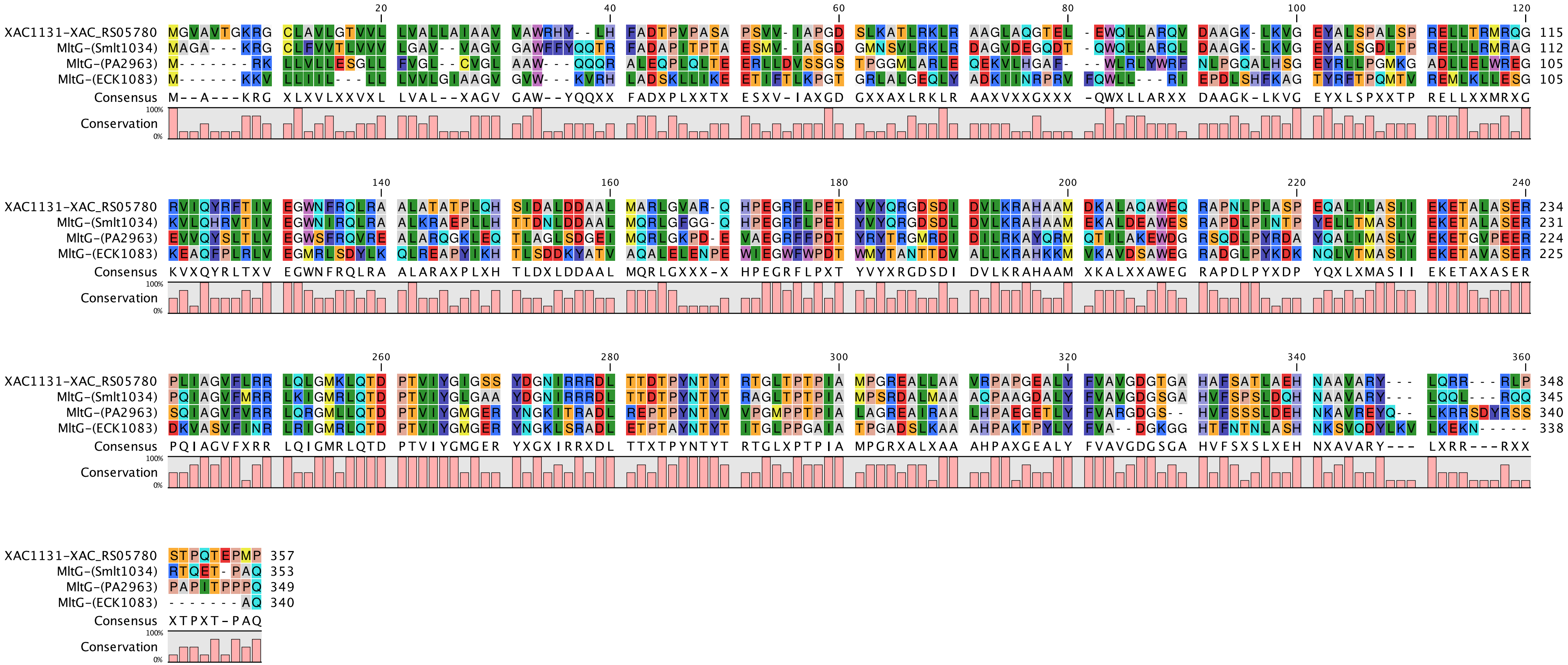

Family 5A - Domain architecture

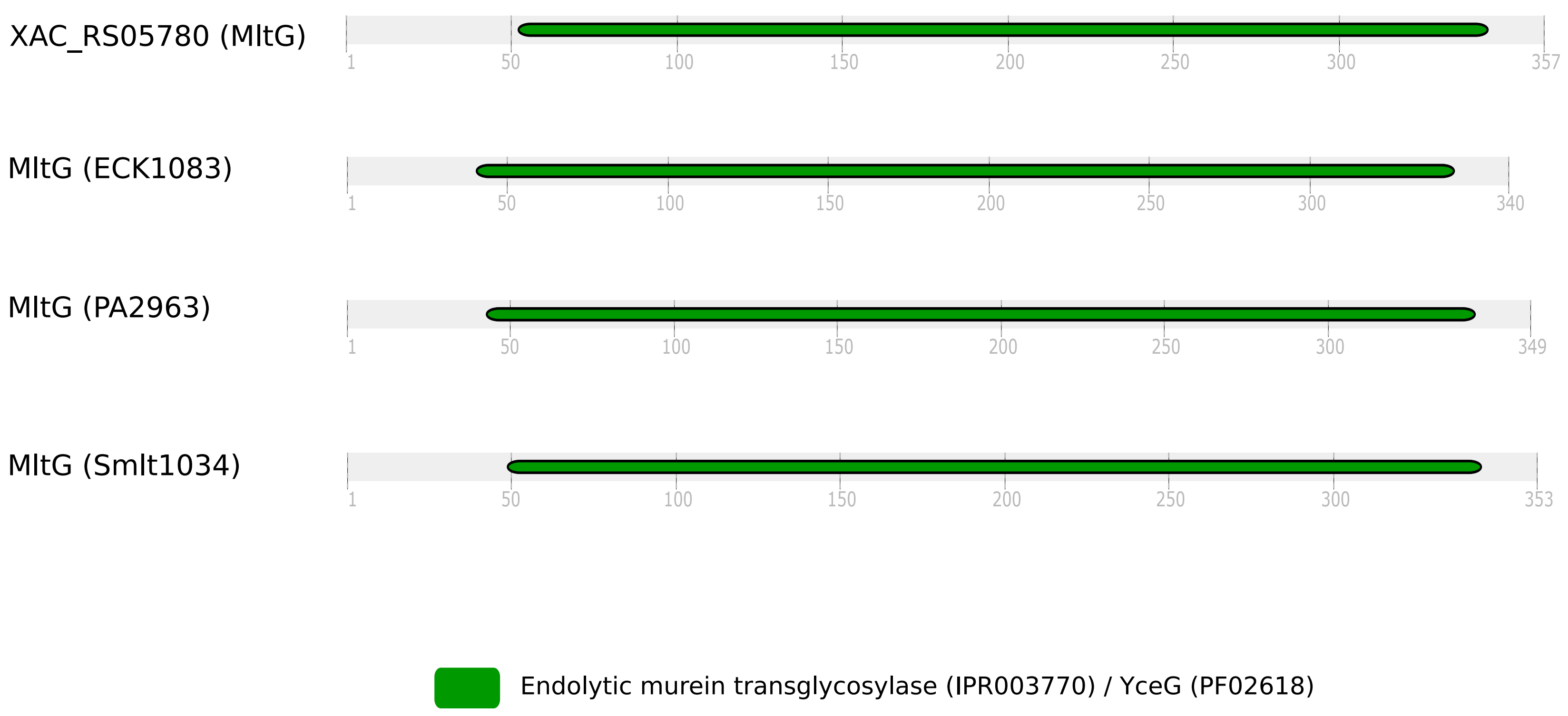

Family 6A - Alignment

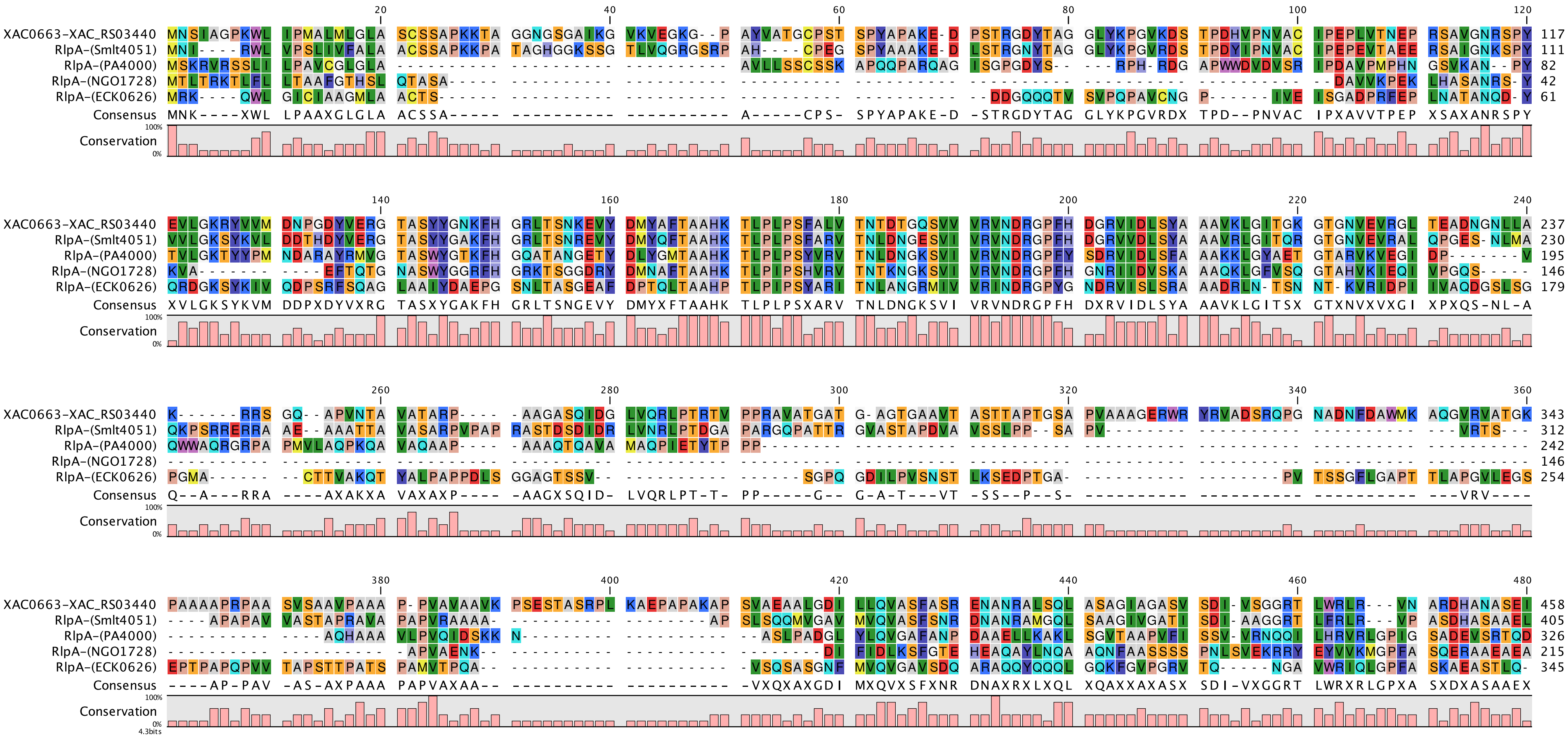

Family 6A - Domain architecture

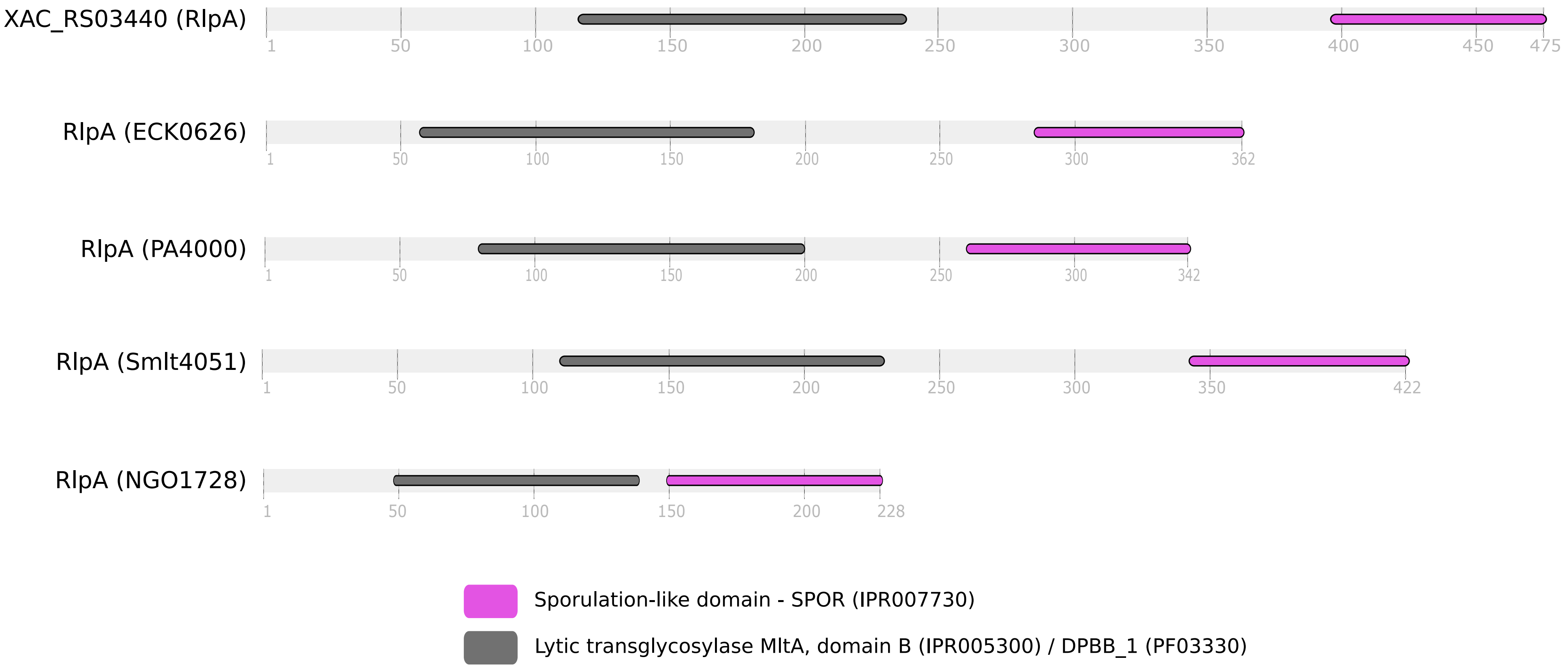

XAC_RS21660 (unknown) - Alignment against Family 3B

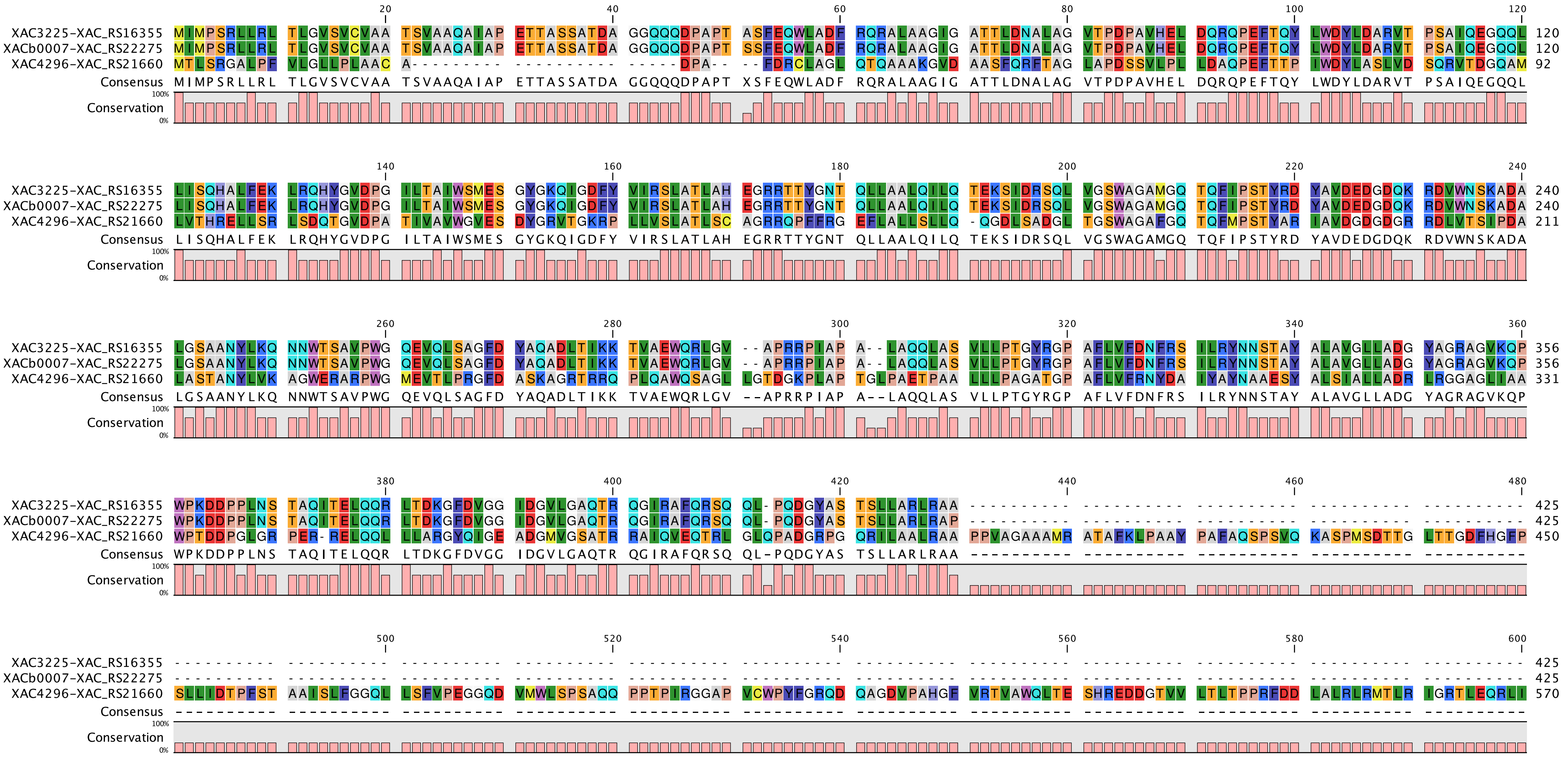

XAC_RS21660 - Domain architecture

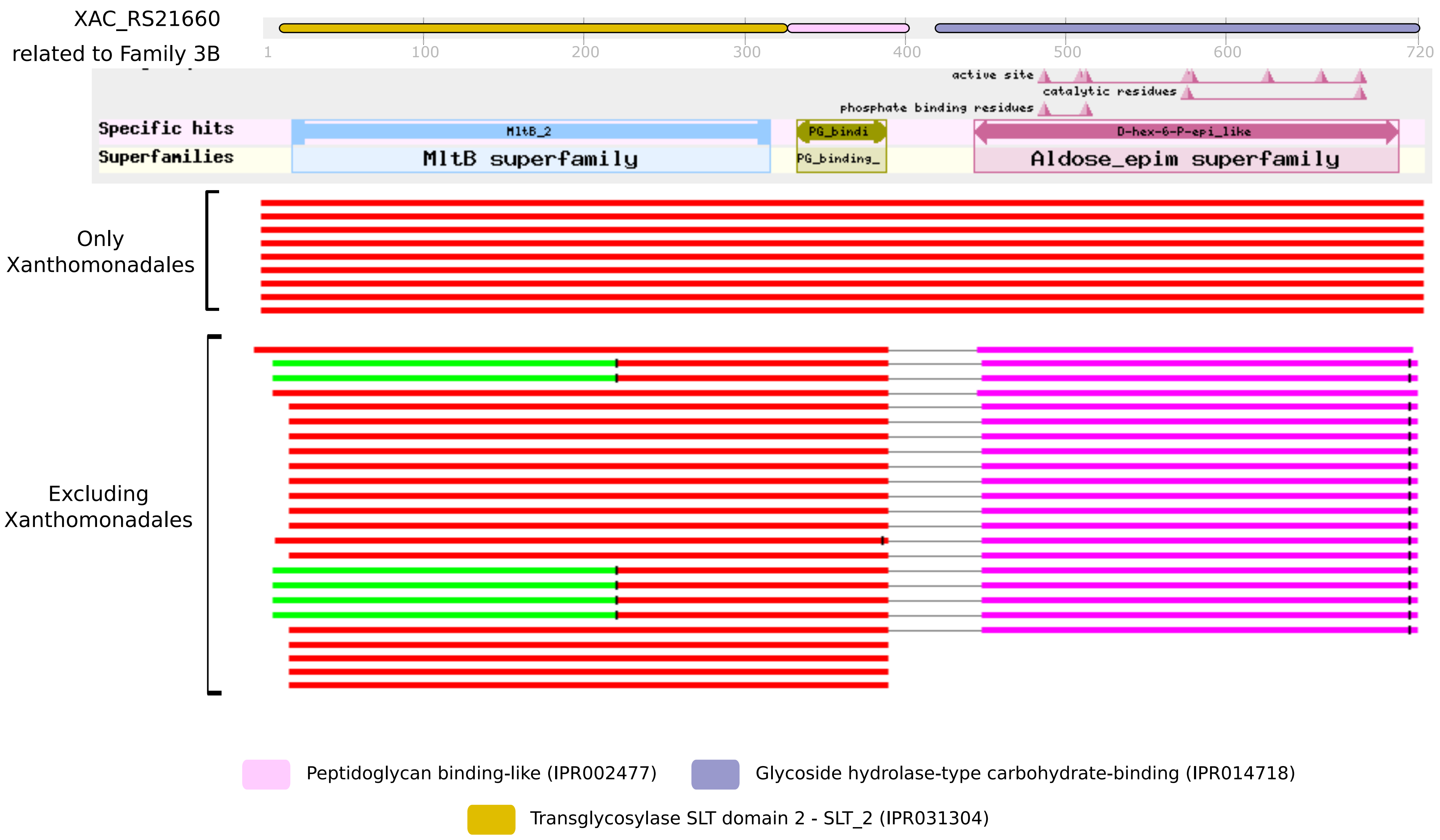

XAC_RS15470 (MtgA)

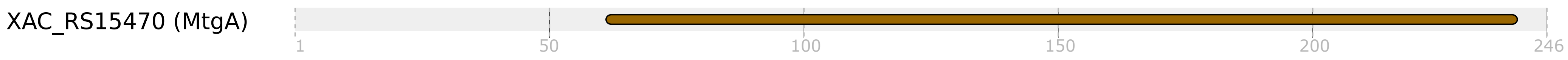

UniProt Reviewed - Q8PI51 - Peptidoglycan polymerase that catalyzes glycan chain elongation from lipid-linked precursors.

Biosynthetic peptidoglycan transglycosylase (IPR011812)
