# Supplementary material for: Transposons and pathogenicity in Xanthomonas: acquisition of murein lytic transglycosylases by TnXax1 enhances Xanthomonas citri subsp. citri 306 virulence and fitness
Source: PeerJ. 2018 Dec 19;6:e6111. doi: 10.7717/peerj.6111 (PMC6304161; doi:10.7717/peerj.6111)
Supplement: Supplemental Information 3 — (M) 1Kb Fermentas DNA Ladder marker. ΔmltB2.1 mutant: (C1) wt mltB2.2 gene, 2,100 bp; (C2) wt mltB2.1 gene, 1,100 bp; (1) wt mltB2.2 gene, 2,100 bp; (2) ΔmltB2.1 mutant, 500 bp; ΔmltB2.2 mutant: (C1) mltB2.2 wt gene, 2,100 bp; (C2) mltB2.1 wt gene, 1,100 bp; (3) ΔmltB2.2, 750 bp; (4) mltB2.1 wt gene, 1,100 bp; ΔmltB2.1-ΔmltB2.2 double mutant: (C1) mltB2.2 wt gene, 2,100 bp; (C2) mltB2.1 wt gene, 1,100 bp; (5) ΔmltB2.2 mutant, 750 bp; (6) ΔmltB2.1, 500 bp.. [file peerj-06-6111-s003.pdf]

M C1 C2 1 2 — *ΔmltB2.1*

M C1 C2 3 4 — *ΔmltB2.2*

M C1 C2 5 6 — *ΔmltB2.1-ΔmltB2.2*

2000

1500

1000

750

500

250
