# Supplementary material for: Transposons and pathogenicity in Xanthomonas: acquisition of murein lytic transglycosylases by TnXax1 enhances Xanthomonas citri subsp. citri 306 virulence and fitness
Source: PeerJ. 2018 Dec 19;6:e6111. doi: 10.7717/peerj.6111 (PMC6304161; doi:10.7717/peerj.6111)
Supplement: Supplemental Information 6 [file peerj-06-6111-s006.docx]

**Table S1. Bacterial strains and plasmids used in this work.**

| **Bacterial strains and plasmids** | **Short name** | **Relevant features** | **Reference** |
| --- | --- | --- | --- |
| *Xantomonas citri* subp. *citri* strain *306* | XccA | Wild strain, citrus pathogen | (da Silva et al. 2002) |
| *ΔmltB2* – chromosome copy | *ΔmltB2.2* | *XAC_RS16355* | This work |
| *ΔmltB2*-pXAC64 | *ΔmltB2.1* | *XAC_RS22275* | This work |
| double mutant: *ΔmltB2-*pXAC64-*mltB2-*chromosome copy | *ΔmltB2.1-mltB2.2* | *XAC_RS22275*, *XAC_RS16355* | This work |
| *E. coli DH5α λpir* | *-* | E44, ΔlacU169 ɸlacΔM15, recA1, endA1, hsdR17, thi-1, gyrA96, relA1, λpir phage lysogen | Biomedal |
| *E. coli SM λpir* | *-* | Km^r^, thi-1, thr, leu, tonA, lacY, sup | Biomedal |
| *E. coli DH10B* | *-* | Δ (*mcrA*, *mcrBC*, *mrr*, A1 and hsdRMS), pir+, *lacZ* for screening | Invitrogen |
| pNPTS138 | - | Suicide vector, LacZ, Km^r^ | (Kaniga 1991) |
| pOK1 | - | Suicide vector, Spec^r^ | (Kaniga 1991) |
| pNPTS138-*ΔmltB2* – chromosome copy | pNPTS138-*ΔmltB2.2* | Suicide vector, LacZ, Km^r^ | This work |
| pOK1-*ΔmltB2-*pXAC64 | pOK1-*ΔmltB2.1* | Suicide vector, Spec^r^ | This work |

da Silva AC, Ferro JA, Reinach FC, Farah CS, Furlan LR, Quaggio RB, Monteiro-Vitorello CB, Van Sluys MA, Almeida NF, Alves LM, do Amaral AM, Bertolini MC, Camargo LE, Camarotte G, Cannavan F, Cardozo J, Chambergo F, Ciapina LP, Cicarelli RM, Coutinho LL, Cursino-Santos JR, El-Dorry H, Faria JB, Ferreira AJ, Ferreira RC, Ferro MI, Formighieri EF, Franco MC, Greggio CC, Gruber A, Katsuyama AM, Kishi LT, Leite RP, Lemos EG, Lemos MV, Locali EC, Machado MA, Madeira AM, Martinez-Rossi NM, Martins EC, Meidanis J, Menck CF, Miyaki CY, Moon DH, Moreira LM, Novo MT, Okura VK, Oliveira MC, Oliveira VR, Pereira HA, Rossi A, Sena JA, Silva C, de Souza RF, Spinola LA, Takita MA, Tamura RE, Teixeira EC, Tezza RI, Trindade dos Santos M, Truffi D, Tsai SM, White FF, Setubal JC, and Kitajima JP. 2002. Comparison of the genomes of two *Xanthomonas* pathogens with differing host specificities. *Nature* 417:459-463. 10.1038/417459a

Kaniga KD, I.; Cornelis, GR . 1991. A wide-host-range suicide vector for improving reverse genetics in Gram-negative bacteria: inactivation of the blaA gene of Yersinia enterocolitica. *Gene- Elsevier* 109: 137-141 10.1016/0378-1119(91)90599-7
